# From rumen to milk: Dietary polyphenols in dairy cows—A critical review

**DOI:** 10.1016/j.vas.2026.100569

**Published:** 2026-01-09

**Authors:** Lucrezia Forte, Nives Parabita, Marta Santoro, Francesco Longobardi, Giuseppe Natrella, John Quiñones, Eric N. Ponnampalam, Igor Tomasevic, Pasquale De Palo, Aristide Maggiolino

**Affiliations:** a Department of Veterinary Medicine, University of Bari Aldo Moro, Strada Provinciale per Casamassima Km 3, Valenzano, Bari 70010, Italy; b Department of Chemistry, University of Bari “Aldo Moro”, Via Orabona 4, 70126 Bari, Italy; c Department of Soil, Plant and Food Science, University of Bari Aldo Moro, Via Amendola 165/a, 70126 Bari, Italy; d Meat Quality Innovation and Technology Centre (CTI-Carne), Universidad de La Frontera, Temuco 4780000, Chile; e Faculty of Agricultural and Environmental Sciences, Universidad de La Frontera, Av. Francisco Salazar 01145, Temuco 4780000, Chile; f School of Agriculture, Food and Ecosystems Sciences, The University of Melbourne, Parkville, VIC 3010, Australia; g Formerly Agriculture Victoria Research, Department of Jobs, Precincts and Regions, Bundoora, VIC 3083, Australia; h Faculty of Agriculture, University of Belgrade, Nemanjina 6, 11080 Belgrade, Serbia; i DIL German Institute of Food Technology, Prof.-von-Klitzing-Str. 7, D-49610 Quakenbrück, Germany

**Keywords:** Dairy cows, Dietary polyphenols, Rumen microbiota, Milk metabolites, Functional dairy products, Bioavailability, Metabolomics

## Abstract

Polyphenols represent a large and structurally diverse family of plant secondary metabolites with bioactive properties. In ruminants, these compounds can influence rumen fermentation, microbial ecology, and nutrient metabolism, offering potential benefits for animal health, productivity, and environmental sustainability. This review synthesizes evidence on the fate of dietary polyphenols in dairy cows from ingestion to their possible secretion into milk. It outlines the main dietary sources and classes of polyphenols, their microbial biotransformations in the rumen, and subsequent host metabolism involving absorption, conjugation, and systemic circulation. Particular attention is given to the mechanisms of mammary uptake and secretion, where most compounds appear as conjugated metabolites such as glucuronides, sulfates, and urolithins rather than parent forms. Although the transfer efficiency from feed to milk is typically low, consistent detection of isoflavone derivatives, phenyl-γ-valerolactones, urolithins, and hippuric acid demonstrates the feasibility of diet-to-milk modulation. Factors affecting bioavailability and transfer include polyphenol structure, dietary matrix, dose, rumen microbiota composition, animal physiology, and feed processing. Advances in high-resolution analytical techniques have improved the characterization of these low-abundance metabolites, yet large variability among studies persists. I*n vivo* studies indicate that polyphenol-derived metabolites in bovine milk occur at low ng/mL to low µg/mL levels, with compounds such as equol, enterolactone, urolithins, phenyl-γ-valerolactones and phenolic acids typically detected in the sub-micromolar range. Overall, dietary polyphenols offer promising opportunities to improve ruminant health and produce milk with enhanced functional quality, but quantitative and mechanistic research is still required to optimize feeding strategies and understand their contribution to milk bioactivity.

## Highlights

• Dietary polyphenols can modulate rumen fermentation and improve cow health.
• Microbial and host metabolism shape polyphenol bioavailability and transfer.
• Polyphenol metabolites like equol and urolithins have been identified in bovine milk.
• Milk enrichment depends on compound type, microbiota, and mammary transport.
• Advanced UHPLC–MS/MS enables precise tracing of milk polyphenol metabolites.

## 1 Introduction

### 1.1 Overview of polyphenols and their significance

Polyphenols constitute a large and structurally diverse class of secondary metabolites widespread across the plant kingdom. A very large number of distinct polyphenol compounds has been identified (Serra et al., 2021). They include phenolic acids, stilbenes, lignans, coumarins, curcumin, tannins and flavonoids (Santhiravel et al., 2022). Each subgroup within the polyphenol cover many subclasses or compounds and they vary in structure and biochemical actions. These compounds, typically bearing multiple hydroxyl groups on aromatic rings, are not directly involved in primary growth or development of plants. Rather, they serve critical ecological functions, including defense against pathogens and herbivores, regulation of plant–pollinator interactions through pigmentation, attract birds and animals over spreading of seeds of weeds, wild plants, and fruits and protection from environmental stressors such as ultraviolet radiation (Bešlo et al., 2022). It has been suggested that a non-trivial fraction of photosynthetically fixed carbon may be channeled to the synthesis of flavonoids—a major subclass of polyphenols—and related phenolic acid compounds (Harun et al., 2025). In the context of animal nutrition, and particularly within dairy production systems, polyphenols have attracted increasing attention due to their diverse bioactive properties. These include strong antioxidant capacity, anti-inflammatory and antimicrobial effects, and the ability to modulate rumen microbial populations and fermentation dynamics (Bešlo et al., 2022; Harun et al., 2025). The incorporation of polyphenol-rich feedstuffs or extracts into ruminant diets has thus emerged as a promising strategy not only for improving animal health and performance, particularly in metabolically stressed, high-yielding dairy cows, but also for promoting environmental sustainability by potentially reducing enteric methane emissions (Harun et al., 2025). Stating these, we also acknowledge here that not all polyphenols are beneficial to animals and their efficiency also vary product to product and a wide range of these compounds can be detrimental to animal productivity and health, when added in the diet above threshold levels. Research in this field increasingly adopts a dual-benefit framework: the improvement of livestock health and productivity on one hand, and the enhancement of dairy product quality on the other. For instance, some polyphenols have demonstrated the capacity to shift rumen fermentation toward more energetically favorable pathways or suppress methanogenic archaea, which can reduce methane outputs (Harun et al., 2025). Furthermore, their antioxidant properties may help mitigate metabolic oxidative stress, thereby improving the physiological resilience, health and welfare of dairy cattle (Harun et al., 2025). Simultaneously, there is growing interest in leveraging dietary polyphenols to enrich the nutritional quality of milk. These interventions aim not only to improve the oxidative stability of milk and dairy products but also to increase the concentration of polyphenols or their bioactive metabolites in milk, thus offering functional food potential for human health (Serra et al., 2021). Nonetheless, the complexity inherent in the term *polyphenol* warrants careful consideration as the physiological and metabolic outcomes associated with one compound or bioactive substance cannot be readily generalized to others (Serra et al., 2021). Different polyphenols can vary substantially in their bioavailability, metabolic transformation, and interaction with the rumen microbiota, all of which influence their absorption, systemic circulation, and eventual secretion into milk (Harun et al., 2025). Consequently, compound-specific and bioactive substance -specific investigations are essential for drawing reliable conclusions and designing effective dietary strategies in dairy systems. Evidence from meta-analyses indicate that tannin-rich polyphenols can reduce enteric methane emissions in ruminants, although responses are dose-, bioactive substance - and diet-dependent (Jayanegara et al., 2012). In addition, endogenous polyphenol oxidase (PPO) in fresh forages can alter protein–polyphenol interactions and reduce proteolysis, with potential downstream effects on nitrogen use efficiency and the fate of polyphenol post-ingestion (Lee, 2014).

### 1.2 Rationale for studying polyphenol transfer to milk

Beyond their impact on animal health and performance, a major rationale for investigating dietary polyphenols in dairy cattle lies in their potential to be transferred—either as intact compounds or as biotransformed metabolites—into milk (Serra et al., 2021). This represents a natural and non-invasive approach for enriching milk with bioactive compounds that may confer health benefits to human consumers. Milk, as a complex emulsion of proteins, fats, and aqueous phases, provides a unique matrix for polyphenol incorporation. Milk proteins, including casein micelles, have been proposed as potential carriers of polyphenols, which may enhance stability and influence bioaccessibility during digestion (Khan et al., 2019). The development of “functional” or “designer” milk, enriched via natural feeding strategies rather than post-harvest fortification, aligns with increasing consumer demand for foods that provide health benefits beyond basic nutrition. Milk enriched in polyphenol compounds or their derivatives may exhibit improved oxidative stability and potentially deliver antioxidant or anti-inflammatory benefits to consumers (Khan et al., 2019; Serra et al., 2021). However, transfer efficiencies are typically low and often compound-specific, which underscores the need for precise analytical approaches and well-controlled feeding trials (Serra et al., 2021), ensuring these compound(s) are transferred to circulatory systems and deposited in animal tissues and reaching milk and meat ultimately. When detectable in bovine milk, polyphenols frequently occur as phase-II conjugates (e.g., glucuronides and sulfates) rather than as aglycones, consistent with extensive biotransformation prior to secretion (Rocchetti et al., 2022). Field and farm-scale studies also show diet-to-milk shifts in the polyphenol profile—for example, the inclusion of grape by-products in small ruminant diets modified milk polyphenol—supporting the feasibility of transfer under practical conditions through dietary means (Bennato et al., 2023). Over the last decade, several reviews have addressed the use of plant polyphenols and related bioactive compounds in ruminant nutrition, often emphasising their effects on animal performance, methane mitigation, oxidative stress and product quality (Ahmed et al., 2024; Bešlo et al., 2022; Besharati et al., 2022; Formato et al., 2022; Harun et al., 2025; Olagaray & Bradford, 2019; Ponnampalam et al., 2022; Serra et al., 2021). While these contributions have provided important insights into the potential of polyphenols as functional feed ingredients, they typically treat the underlying metabolic pathways and the feed-to-milk transfer of polyphenols and their metabolites only briefly, or in a more generic ruminant context. To date, no review has systematically followed the entire continuum “from rumen to milk” in dairy cows, integrating microbial and host metabolism with mammary transport processes, detailed metabolite profiling in milk, and implications for functional dairy products.

### 1.3 Objectives of the review

The overarching aim of this article is to provide a critical and integrated appraisal of how dietary polyphenols are transformed and transferred along the continuum from rumen to bovine milk. In contrast to previous reviews that have mainly focused on production responses or broad aspects of product quality in ruminants, our specific contribution is to link (i) the metabolic transformations of different polyphenol classes in the rumen and post-ruminal compartments, (ii) the systemic fate and mammary transport mechanisms of key microbial- and host-derived metabolites, and (iii) the analytical strategies used to detect these compounds in milk, with (iv) the implications for developing polyphenol-enriched or otherwise functionally enhanced dairy products.

Although the broader implications for human health provide an important underlying motivation, the central focus remains on characterizing compositional changes in milk resulting from polyphenol-rich dietary interventions. Given the typically low transfer and strong matrix effects, we will also consider analytical advances—such as chemical-isotope-labelling LC–MS targeting the amine/phenol submetabolome—that enhance coverage and quantification in milk (Mung & Li, 2017). In addition to providing an updated and comprehensive overview, this review explicitly aims to critically appraise the available evidence. Rather than only summarizing individual studies, we highlight inconsistencies among findings, methodological limitations and gaps, and mechanistic uncertainties along the continuum from rumen metabolism to milk secretion. Where data are lacking or conflicting, we propose working hypotheses and priorities for future research to support more rational use of dietary polyphenols in dairy feeding systems. To guide the reader, the review is organised into five main thematic sections. First, we summarise the sources and classification of dietary polyphenols relevant to dairy feeding systems (Section 3). Second, we describe the metabolic transformations of these compounds in the rumen and post-ruminal compartments (Section 4). Third, we examine milk transfer mechanisms and the metabolites that have been identified in bovine milk (Section 5), followed by a discussion of factors influencing bioavailability and transfer (Section 6) and analytical advances for their quantification (Section 7). Finally, we address implications for milk quality and potential human health benefits, and outline future research directions (8, 3.3, 4). By combining mechanistic insights with evidence from in vivo trials and emerging metabolomics studies in milk (e.g., Agulló et al., 2024; Bennato et al., 2023; Connolly et al., 2023; Rocchetti et al., 2022), this review ultimately aims to identify realistic opportunities and current limitations for leveraging dietary polyphenols in precision feeding and the design of functional dairy products.

## 2 Review method

To ensure the accuracy and comprehensiveness of this review, an extensive bibliographic investigation was conducted across several scientific databases, including Scopus, Google Scholar, and PubMed, between January and June 2025. The search strategy was designed to capture both foundational and recent advancements concerning the metabolism, bioavailability, and transfer of dietary polyphenols in dairy cows, with a particular focus on their presence and transformation from feed to milk. The initial search targeted the core concepts of polyphenols in ruminant nutrition, rumen metabolism, and milk bioactive composition. Subsequently, the focus was refined to include specific metabolic pathways, mammary transfer mechanisms, and analytical methodologies for detecting polyphenols and their metabolites in milk. The primary search terms included combinations of: “dietary polyphenols”, “dairy cows”, “rumen metabolism”, “milk composition”, “polyphenol transfer”, “bioavailability”, “milk metabolites”, “flavonoids”, “isoflavones”, “tannins”, “phenolic acids”, “urolithins”, “phenyl-γ-valerolactones”, “antioxidant activity”, and “milk enrichment”. Boolean operators “AND” and “OR” were applied to connect key concepts and ensure a comprehensive yet targeted selection of records. Representative queries included, for example: “(dairy cows OR ruminants) AND (dietary polyphenols OR flavonoids OR tannins) AND (milk composition OR metabolite transfer)” and “(polyphenols OR isoflavones) AND (rumen metabolism OR mammary secretion)”. The inclusion criteria for the selection of publications were as follows: Direct relevance to the topic, i.e., studies examining the digestion, metabolism, or milk transfer of dietary polyphenols in ruminants; Publication date between 2005 and 2025, ensuring coverage of the most recent and methodologically robust research and scientific quality and impact. Peer-review status was defined as publication in a scientific journal implementing a formal external peer-review process. Articles in peer-reviewed journals constituted the core body of evidence included in the main synthesis, whereas non–peer-reviewed sources (e.g., theses, technical reports) were used only when they provided unique contextual or methodological information and are clearly identified as such in the text. Older references were included when they provided historical context or seminal findings relevant to understanding rumen transformations, polyphenol classification, or analytical development in milk metabolomics. No a priori numerical threshold based on citation counts or impact factor was applied for study exclusion, in order to avoid penalizing recent or highly specialized contributions. The potential risk of bias in individual studies was appraised qualitatively, considering study design (presence of a control group, randomization or cross-over design where applicable), sample size, diet characterization, study duration, and the appropriateness and validation of analytical methods for polyphenol detection. Owing to the heterogeneity of experimental designs and outcomes, no single standardized risk-of-bias tool could be consistently applied. References were managed using EndNote™ (Clarivate Analytics) for systematic organization and to avoid duplication. The selection process adhered to PRISMA guidelines for narrative reviews, emphasizing transparency in literature inclusion. Review and research articles, book chapters, and thesis were all considered where they contributed novel or comprehensive insights into the metabolic pathways and detection of polyphenols in dairy systems.

**Fig. 1:**
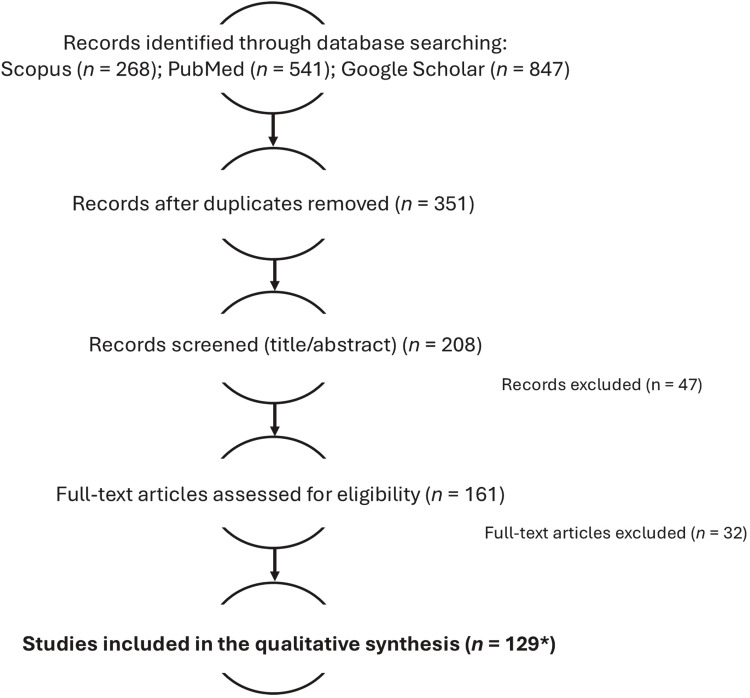
PRISMA-style flow diagram of the literature search and study selection process. The figure shows the number of records identified in each database, the removal of duplicates, the number of records screened at title/abstract level, full-text articles assessed for eligibility, reasons for exclusion, and the final number of studies included in the qualitative synthesis.

The overall selection process is summarized in a PRISMA-style flow diagram (Fig. 1), which reports the number of records identified in each database, duplicates removed, records screened at title/abstract level, full-text articles assessed for eligibility, reasons for exclusion, and the final number of studies included in the qualitative synthesis. A small number of seminal or historical references published before 2005 were additionally identified from reference lists and included to provide essential background on rumen microbiology, polyphenol metabolism, and analytical developments.

## 3 Sources and classification of dietary polyphenols in dairy systems

Polyphenol compounds are ubiquitous in the plant kingdom, and their presence in dairy cattle diets arises from a wide spectrum of natural feeds, specialized forages, supplemented grains and seeds, agro-industrial by-products, and targeted plant extracts. Understanding the diversity and classification of these compounds is fundamental for anticipating their metabolic transformations and potential transfer into milk. In practice, both the dose and the matrix (whole feed vs. extract) strongly influence ruminal transformations and downstream availability for transfer (Jayanegara et al., 2012; Lee, 2014)

### 3.1 Classification of polyphenols

Polyphenols are typically grouped based on their chemical structure into flavonoids, non-flavonoids, and, often treated separately due to their polymeric structure and functional properties, (Serra et al., 2021). For more details on phytochemicals, their subclasses and structure, refer to Santhiravel et al. (2022).

• Flavonoids represent the largest class of plant polyphenols, with over 4000 known compounds, all sharing a C6–C3–C6 carbon skeleton arranged into three rings (A, B, and C). These low-molecular-weight secondary metabolites are synthesized from aromatic amino acids and are subdivided into several relevant subclasses for their relevance to human and animal nutrition (Bešlo et al., 2022):
− Flavonols (e.g., quercetin, kaempferol): Commonly found in fruit skins, vegetables, and grasses.
− Flavones (e.g., apigenin, luteolin): Present in herbs and cereal grains.
− Flavanols (or flavan-3-ols; e.g., catechin, epicatechin, epigallocatechin [EGC], epigallocatechin gallate [EGCG]): Abundant in tea and grape derivatives.
− Flavanones (e.g., hesperetin, naringin): Typically found in citrus fruits.
− Anthocyanins (e.g., cyanidin-3-glucoside, malvidin-3-glucoside): Natural pigments conferring red, purple, and blue hues to fruits and flowers.
− Isoflavones (e.g., genistein, daidzein, formononetin, biochanin A): Phytoestrogenic compounds primarily found in legumes such as soybeans and red clover.
• Non-Flavonoids comprise several structurally distinct polyphenol families (Serra et al., 2021):
− Phenolic acids, which include hydroxybenzoic acid derivatives (e.g., gallic acid) and hydroxycinnamic acid derivatives (e.g., caffeic, ferulic, p-coumaric, sinapic acids).
− Stilbenes, characterized by a C6-C2-C6 structure, of which resveratrol (from grapes) is the most widely studied.
− Lignans, such as secoisolariciresinol and matairesinol, are primarily found in flaxseed. Dietary lignans can be converted to the mammalian lignan enterolactone, which may appear in milk (Brito & Zang, 2018), which offer positive carcinogenic effects.
• Tannins, defined by their ability to precipitate proteins, are often treated separately. They are classified into:
− Hydrolyzable tannins: Comprising gallotannins and ellagitannins, which are esters of gallic or ellagic acid with a polyol core (commonly glucose), and can be broken down by acids, bases, or enzymes (Serra et al., 2021).
− Condensed tannins (proanthocyanidins): Non-hydrolyzable polymers of flavan-3-ol units, more resistant to degradation and structurally included within the flavonoid family (Powell et al., 2009; Serra et al., 2021). Their nutritional impact is biphasic: excessive levels can depress intake, digestibility and animal productivity, whereas low–moderate levels can protect dietary protein in the rumen and modulate methane mitigation (Jayanegara et al., 2012; Naumann et al., 2017).

### 3.2 Common polyphenol sources in dairy diets

Polyphenols can enter the ruminant diet through various channels:

• Natural Forages:
ο Grasses: Pasture grasses provide baseline levels of polyphenols, particularly flavonols like quercetin and kaempferol. Mountain pastures are noted as richer sources (Bešlo et al., 2022).
ο Legumes: Among the most important polyphenol sources, red clover (Trifolium pratense) is particularly rich in isoflavones and polyphenol oxidase (PPO) activity (Bešlo et al., 2022). Upon tissue damage and during ensiling, PPO catalyses o-diphenol oxidation to o-quinones that bind proteins, reducing proteolysis and altering phenol availability (Lee, 2014).
ο Other Forages: Chicory contains various phytoestrogens (Bešlo et al., 2022). Additional sources include sulla-based pastures, lentil straw, Atriplex and olive leaves, and silages made from paulownia (Harun et al., 2025). Olive-derived forages/by-products contribute polyphenols such as oleuropein and hydroxytyrosol; inclusion levels and processing markedly affect their bioactivity (Tzamaloukas et al., 2021).
• Grains and Seeds:
ο Soybean meal: A primary protein supplement and rich source of isoflavones like genistein and daidzein Bešlo et al. (2022).
ο Grape seed and grape seed extract (GSE): Provide high concentrations of proanthocyanidins and monomeric flavan-3-ols (catechin, epicatechin) (Bešlo et al., 2022).
ο Linseed (flaxseed): Commonly used for its fatty acid profile, but also a key source of lignans (Harun et al., 2025). Increasing flaxseed meal can raise milk enterolactone in dairy cows, evidencing diet-to-milk transfer of lignan metabolites (Brito & Zang, 2018; Ghedini et al., 2017).
ο Other seeds: Sesame, cocoa bean shells, cumin seed extract, and coix seed also contribute a variety of polyphenols (Harun et al., 2025). Cocoa bean shell is a by-product particularly rich in phenolics (including catechins) and methylxanthines, already tested in small ruminants (Rojo-Poveda et al., 2020; Renna et al., 2022).
• Agro-Industrial By-Products: These are increasingly valued for their economic and environmental advantages, aligning with sustainability and circular economy goals in agriculture (Serra et al., 2021) and animal production (Ponnampalam et al., 2025).
ο Grape pomace/marc: A rich source of both condensed and hydrolyzable tannins, flavonoids, and phenolic acids, depending on grape type and processing (Serra et al., 2021). Feeding studies indicate shifts in milk phenolics with grape pomace inclusion, supporting practical transfer (Bennato et al., 2023).
ο Citrus pulp: Derived from juice production, containing flavanones such as hesperidin and naringin Serra et al. (2021). These flavanones are well-documented constituents of citrus by-products used in ruminant feeding (Santos et al., 2014).
ο Olive pomace and olive leaves: By-products from olive oil production, rich in phenolics such as oleuropein, hydroxytyrosol, and tyrosol (Harun et al., 2025). Reviews underscore variable inclusion limits and technological treatments to manage fibre/lipid load while preserving phenolics (Tzamaloukas et al., 2021).
ο Other by-products: Include tomato pomace, pomegranate peel, wine lees (fermented or unfermented), and mango meal (Harun et al., 2025).
ο Propolis-derived products: Containing a wide array of phenolic compounds, occasionally used as feed supplements (Harun et al., 2025). Composition is highly source-dependent, which complicates dose standardisation.
• Plant Extracts:
ο Green tea extract (GTE): A highly concentrated source of catechins, especially EGCG, EGC, and EC (Bešlo et al., 2022).
ο Other extracts: Rosemary, citrus, and marigold extracts have also been explored for their polyphenolic profiles (Bešlo et al., 2022). Citrus flavonoid concentrates (naringin/hesperidin) have documented antimicrobial and immunomodulatory effects relevant to dairy systems (Yang et al., 2022; Zhao et al., 2023).

The valorization of agro-industrial by-products not only reduces feed costs and waste but also offers functional benefits for animal health and milk quality (Grasso et al., 2024). Their polyphenolic richness supports their inclusion in dairy rations aimed at biofunctional milk production (Serra et al., 2021). Nevertheless, anti-nutritional thresholds (notably for tannins) and the presence of other co-constituents (lipids, fibre, alkaloids) require careful formulation and monitoring (Naumann et al., 2017).

### 3.3 Factors affecting polyphenol content in feedstuffs

The polyphenol content in feed ingredients is subject to substantial variability, shaped by multiple interrelated factors:

• Type of pastures and fodders:
• Genetic factors: Plant species and cultivar determine the baseline polyphenol profile (Serra et al., 2021).
• Harvest maturity: The stage at which plants are harvested influences both the quantity and quality of polyphenols (Serra et al., 2021).
• Environmental conditions: Soil type, nutrient availability, light intensity, temperature, and rainfall all modulate secondary metabolite synthesis in plants (Serra et al., 2021).
• Processing and storage: Polyphenol levels are significantly affected by post-harvest practices such as drying, pelleting, ensiling, and extraction. Exposure to heat, oxygen, light, or enzymatic oxidation (e.g., PPO activity) can lead to polymerization or degradation of polyphenols (Lee, 2014; Serra et al., 2021). For instance, red clover’s endogenous PPO becomes active upon tissue damage, oxidizing o-diphenols into o-quinones that can bind proteins, reducing free phenol availability (Lee, 2014). Ensiling high-PPO forages can improve nitrogen use efficiency and alter the speciation of extractable phenolics available post-rumen (Lee, 2014).

Such variability poses a major challenge for formulating reliable polyphenol-based dietary interventions. Merely selecting a feed labeled as “polyphenol-rich” does not guarantee a consistent dose or profile of bioactive compounds. The availability of individual compounds including their subclasses in the feed(s) must be mentioned along with the quantity or concentrations. Therefore, robust analytical methods and standardized processing protocols are necessary to ensure reproducibility in polyphenol supplementation strategies and the expected functional outcomes in animals.

A particularly illustrative case is that of tannins. Although high concentrations can impair nutrient digestibility and reduce feed intake thereby reducing animal performance and productivity. A low to moderate levels of condensed tannins (e.g., 20–45 g/kg dry matter) are increasingly recognized for their potential benefits, such as improving protein utilization by protecting it from ruminal degradation and reducing enteric methane emissions (Powell et al., 2009). Contemporary reviews corroborate this biphasic response and emphasize tailoring source and dose to the basal diet (Jayanegara et al., 2012; Naumann et al., 2017). A summary of major polyphenol classes and their dietary sources in dairy cattle is provided in Table 1.

**Table 1:** Major dietary polyphenol classes, representative compounds and sources in dairy cattle feed. Overview of the main polyphenol classes and subclasses relevant to dairy systems, including representative compounds, common primary feed sources and major agro-industrial by-products, with key references.

Polyphenol Class	Sub-class	Key Compounds Example(s)	Common Feed Sources	By-product	References
Flavonoids	Isoflavones	Genistein, Daidzein, Formononetin, Biochanin A	Soy products (meal, beans), Red clover, Lucerne, White clover	No	(Bešlo et al., 2022)
	Flavan-3-ols (Catechins)	(+)-Catechin, (-)-Epicatechin, EGCG, EGC	Green tea extract, Grape seed/pomace, Cocoa shells, Berries, Apples	Yes	(Rojo-Poveda et al., 2020; Bešlo et al., 2022)
	Flavonols	Quercetin, Kaempferol	Onions, Kale, Broccoli, Apples, Berries, Grasses	No	(Bešlo et al., 2022)
	Flavones	Apigenin, Luteolin	Parsley, Thyme, Celery, Cereals, Herbs	No	(Bešlo et al., 2022)
	Flavanones	Hesperetin, Naringenin	Citrus pulp (fresh/dried), Citrus peel	Yes	(Bešlo et al., 2022)
	Anthocyanins	Cyanidin; Delphinidin (class pigments)	Berries, Red grapes (*limited in standard dairy diets*)	No	(Bešlo et al., 2022)
	Proanthocyanidins (Condensed Tannins)	Polymers of catechin/epicatechin	Grape seed/pomace, Birdsfoot trefoil, Sulla forage, Tree bark	Yes	(Bešlo et al., 2022)
Non-Flavonoids	Phenolic Acids (Hydroxybenzoic)	Gallic acid	Nuts, Tannin-rich forages, Fruits	No	(Serra et al., 2021)
	Phenolic Acids (Hydroxycinnamic)	Caffeic acid, Ferulic acid, p-Coumaric acid, Sinapic acid	Grasses, Cereals, Coffee by-products, Fruits and vegetables	Yes	(Serra et al., 2021)
	Stilbenes	Resveratrol	Grapes, Grape pomace	Yes	(Serra et al., 2021)
	Lignans	Secoisolariciresinol, Matairesinol	Flaxseed (linseed), Sesame, Other oilseeds and cereals	No	(Serra et al., 2021)
Tannins	Hydrolyzable Tannins	Gallotannins, Ellagitannins	Oak leaves, Chestnut, Pomegranate peel, Nuts	No	(Serra et al., 2021)
	Condensed Tannins	(Same as Proanthocyanidins)	(See above)	Yes	(Powell et al., 2009; Serra et al., 2021)
Others	Olive-derived Polyphenols	Oleuropein, Hydroxytyrosol, Tyrosol	Olive cake/pomace, Olive leaves	Yes	(Tzamaloukas et al., 2021; Harun et al., 2025)
	Bee-derived Compounds	Caffeic acid phenethyl ester (CAPE), Flavonoid mix	Propolis-based products	Yes	(Harun et al., 2025)
	Grape pomace in milk studies	Mixed flavonoids; Phenolic acids; Tannins	Grape pomace/marc	Yes	(Bennato et al., 2023)

EGCG = epigallocatechin gallate; EGC = epigallocatechin.

## 4 Metabolic transformations of dietary polyphenols in dairy cows

**Fig. 2:**
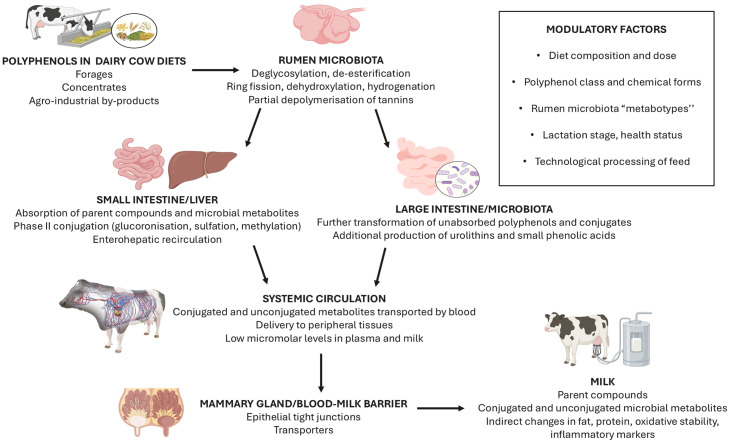
Conceptual overview of the “from rumen to milk” continuum for dietary polyphenols in dairy cows.

The metabolic fate of dietary polyphenols in dairy cows is a multifaceted process involving extensive bio-transformations, predominantly initiated by the rumen microbiota and subsequently passed through digesta, absorbed in the gastrointestinal tract and deposited in host tissues. In ruminants, the presence of the rumen introduces variables that differ markedly from monogastric species, as microbial bio-transformations precede host absorption and frequently determine the nature of circulating and ended as milk-borne metabolites (Aguiar et al., 2014). As a result, the chemical species that reach the absorptive epithelia—and are later detected in plasma and milk—are often conjugated or microbially derived catabolites rather than the natural dietary forms (Mena et al., 2019; Wein et al., 2016). Fig. 2 provides a schematic overview of the entire pathway of polyphenols, from dietary intake to their appearance in milk.

### 4.1 Rumen metabolism – the primary site of transformation

The rumen is the principal site for the initial transformation of dietary polyphenols in ruminants. The resident microbiota, together with ruminal conditions (pH, retention time, redox status), promotes a series of reactions including deglycosylation, de-esterification, ring fission, dehydroxylation and hydrogenation, which profoundly reshape the structures and bioactivity of parent compounds (Bešlo et al., 2022; Cheng et al., 1969; McSweeney & Mackie, 1997; O’Donovan & Brooker, 2001; Simpson et al., 1969).

Isoflavones. Isoflavone glycosides from legumes (e.g., formononetin, biochanin A) are rapidly deglycosylated to yield daidzein and genistein, which are further reduced and dehydroxylated by specific rumen bacteria, leading to the formation of the mammalian lignans equol and O-desmethylangolensin (O-DMA) (Daems et al., 2016; Dadáková et al., 2020; Ghedini et al., 2017; McSweeney & Mackie, 1997).

Flavan-3-ols. Monomeric flavan-3-ols (catechin, epicatechin) and oligomeric procyanidins are subject to microbial ring fission and stepwise degradation to phenyl-γ-valerolactones and phenylvaleric acids, which represent key intermediates on the route to smaller phenolic acids (Mena et al., 2019; Wein et al., 2016). Depending on dose and matrix, a fraction of flavan-3-ols may escape extensive ruminal degradation, contributing to post-ruminal absorption of intact or partially transformed species.

Flavonols. Flavonols such as quercetin undergo deglycosylation followed by deconjugation, ring fission and dehydroxylation, generating a variety of phenylacetic and phenylpropionic acids. In vivo data in cows suggest that ruminal degradation of quercetin is substantial, with only a limited amount of the parent compound reaching the small intestine (Berger et al., 2015).

Tannins. Hydrolysable tannins are cleaved into gallic or ellagic acid and their sugar cores, which are further metabolised by rumen microbes to urolithins and other low-molecular-weight derivatives (Espín et al., 2013; González-Barrio et al., 2012). Condensed tannins (proanthocyanidins) are more resistant to complete depolymerisation, but can still undergo partial cleavage and structural modification; at the same time, their strong protein-binding capacity reduces ruminal protein degradation and alters nitrogen partitioning (Herremans et al., 2020; Jayanegara et al., 2012; Naumann et al., 2017).

Phenolic acids. Phenolic acids may be ingested directly or arise as downstream metabolites of more complex polyphenols. In the rumen they are mainly subjected to reduction and dehydroxylation, leading to simpler benzoic and phenylpropionic acids, although complete mineralisation of the aromatic ring appears limited under typical ruminal conditions (González-Barrio et al., 2012; Simpson et al., 1969).

**Fig. 3:**
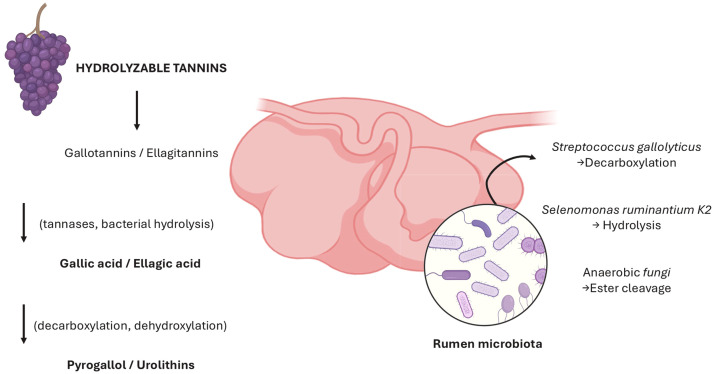
Interaction between hydrolysable tannins and enzymatic pathways and microbial actors in the rumen.

A schematic representation of these ruminal transformation pathways for the main dietary polyphenol classes is provided in Fig. 3, while Table 2 summarises the key reactions and predominant microbial-derived metabolites identified in ruminants.

**Table 2:** Overview of rumen and post-ruminal metabolic pathways of major dietary polyphenol classes in dairy cows.

Polyphenol class / exemplar compounds	Main rumen transformations	Key microbial-derived metabolites	Post-ruminal fate (absorption, conjugation, further metabolism)	References
Isoflavones (e.g., formononetin, daidzein, genistein)	Rapid deglycosylation of plant glycosides; reduction, dehydroxylation and ring fission by specialised rumen bacteria.	Daidzein, genistein, O-desmethylangolensin (O-DMA), equol and other mammalian lignans.	Absorption of aglycones and microbial metabolites in the small intestine; extensive phase II conjugation (glucuronides, sulfates); systemic distribution and partial appearance in milk as free and conjugated forms.	McSweeney and Mackie (1997); Yuan et al. (2007); Daems et al. (2016); Dadáková et al. (2020); Ghedini et al. (2017); Brito and Zang (2018); Křížová et al. (2021).
Flavan-3-ols and procyanidins (e.g., catechin, epicatechin, grape seed proanthocyanidins)	Partial depolymerisation of oligomers; ring fission, dehydroxylation and hydrogenation of monomers in the rumen.	Phenyl-γ-valerolactones, phenylvaleric acids and downstream simple phenolic acids; residual intact catechins at higher doses or in protective matrices.	Absorption of phenyl-γ-valerolactones, phenylvaleric acids and some catechins; conjugation in enterocytes and liver; further metabolism to smaller phenolic acids; limited transfer to milk at low concentrations.	Wein et al. (2016); Mena et al. (2019); Huang et al. (2019); Rocchetti et al. (2022); Bennato et al. (2023).
Flavonols (e.g., quercetin, rutin)	Deglycosylation of rutin and other glycosides; extensive degradation of quercetin aglycone in the rumen (ring fission, dehydroxylation).	Dihydroxy- and trihydroxy-phenylacetic and phenylpropionic acids; 3,4-dihydroxyphenylacetic acid and related derivatives.	Limited escape of intact quercetin to the small intestine; absorption mainly as microbial phenolic acids; rapid conjugation and systemic distribution; very low recovery of parent compound in milk.	Berger et al. (2015); Carrasco-Pozo et al. (2015); Lewandowska et al. (2013); Manach et al. (2004); D’Archivio et al. (2010).
Hydrolysable tannins (e.g., ellagitannins, gallotannins)	Hydrolysis of ester bonds releasing gallic and ellagic acids; further microbial transformation of ellagic acid.	Urolithins (e.g., urolithin A, B and related derivatives), simple phenolic acids and other low-molecular-weight metabolites.	Absorption of urolithins and phenolic acids in the intestine; extensive conjugation; enterohepatic recycling; potential low-level secretion into milk as conjugated forms.	González-Barrio et al. (2012); Espín et al. (2013); Tomás-Barberán et al. (2014).
Condensed tannins (proanthocyanidins)	Partial depolymerisation and structural modification in the rumen; strong binding to dietary and salivary proteins, reducing ruminal proteolysis.	Limited formation of identifiable monomeric flavan-3-ols and phenolic acids; most high-molecular-weight structures remain unabsorbed.	Unabsorbed tannins and complexes pass to the lower gut; indirect systemic effects via modulation of rumen fermentation, nitrogen partitioning and microbial populations rather than direct absorption of intact polymers.	Jayanegara et al. (2012); Naumann et al. (2017); Herremans et al. (2020); Olagaray and Bradford (2019); Bešlo et al. (2022); Ahmed et al. (2024).
Phenolic acids and other simple phenolics (e.g., caffeic, ferulic, p-coumaric acids)	Reduction, dehydroxylation and decarboxylation of aromatic acids; interconversion with upstream flavonoid-derived metabolites.	Benzoic acids, phenylpropionic and phenylacetic acids, dihydrocaffeic acid and related compounds.	Absorption along the intestine as small phenolic acids; extensive phase II conjugation; systemic circulation at low micromolar or sub-micromolar concentrations; limited but potentially biologically relevant delivery to the mammary gland.	Lafay and Gil-Izquierdo (2008); D’Archivio et al. (2010); Lewandowska et al. (2013); Bohn (2014); Teng and Chen (2019); Zieniuk (2023).

O-DMA = O-desmethylangolensin; PVL = phenyl-γ-valerolactone.

Overall, the metabolic activity of rumen microbiota produces a dramatically altered profile of polyphenol compounds compared to the ingested forms, and this bio-transformation is central to understanding the systemic availability and biological activity of these metabolites. Additionally, dietary polyphenols can modulate ruminal fermentation parameters:

• Certain compounds, notably flavonoids and tannins, are associated with reduced methane emissions, potentially offering both environmental and nutritional benefits (Bešlo et al., 2022; Harun et al., 2025). Mechanistically, tannins can shift microbial consortia and hydrogen sinks, but responses vary with dietary source and dose rate (Jayanegara et al., 2012; Herremans et al., 2020).
• Polyphenols can alter the volatile fatty acid (VFA) profile, typically decreasing the acetate:propionate ratio, which may have implications for energy metabolism in the host (Wang et al., 2021). Multiple trials with plant polyphenol sources report lower A:P and higher propionate—consistent with modest shifts toward more energy-efficient fermentation—though effects are not universal (Jalal et al., 2025).
• Through their protein-binding activity, tannins reduce protein degradation and consequently lower ruminal ammonia concentrations, improving nitrogen retention if post-ruminal digestion is efficient (Aguiar et al., 2014). Meta-analytic evidence indicates limited or no average effect of tannins on milk yield/composition at practical doses despite improved N partitioning (Herremans et al., 2020).

Notably, inter-animal variability (“metabotypes”) in microbial composition and function can produce substantial differences in the formation of equol (from isoflavones) or urolithins (from ellagitannins), even under identical diets—adding uncertainty to expected metabolite levels in milk (Dadáková et al., 2020; González-Barrio et al., 2012).

### 4.2 Post-ruminal metabolism (small intestine, liver, large intestine)

Polyphenol aglycones and microbial-derived metabolites that escape degradation or absorption in the rumen reach the small intestine, where further absorption and biotransformation occur. In line with monogastric models, intestinal uptake is generally favoured for relatively small and moderate lipophilic molecules, whereas highly polar or extensively polymerised structures show limited permeability (Bohn, 2014; Lewandowska et al., 2013; Manach et al., 2004). Once absorbed, these compounds undergo extensive phase II conjugation (glucuronidation, sulfation and methylation) in enterocytes and hepatocytes, leading to a wide array of circulating conjugated metabolites rather than free aglycones (D’Archivio et al., 2010; Lafay & Gil-Izquierdo, 2008; Teng & Chen, 2019). Conjugated metabolites and residual microbial products can then be redistributed via the systemic circulation to peripheral tissues, including the mammary gland, or excreted in bile and urine. Enterohepatic recirculation, involving biliary secretion of conjugates followed by deconjugation and reabsorption in the distal gut, further prolongs their residence time in the body (D’Archivio et al., 2010; Espín et al., 2013; Manach et al., 2004). In dairy cows, in vivo studies with model compounds such as catechins and ellagitannin-derived metabolites confirm that only a small fraction of ingested polyphenols appears in plasma as identifiable entities, and even less reaches milk (González-Barrio et al., 2012; Wein et al., 2016). In parallel, unabsorbed polyphenols and conjugates that reach the large intestine can be further transformed by hindgut microbiota, generating additional pools of low-molecular-weight metabolites that may contribute to systemic exposure over longer time scales (Espín et al., 2013; Makarewicz et al., 2021). A concise overview of the main post-ruminal fates of dietary polyphenols and their microbial products in dairy cows, together with predominant circulating and excreted metabolites, is provided in Table 2. Although the main metabolic routes of dietary polyphenols in ruminants are now relatively well described, several important uncertainties remain when these pathways are extrapolated to dairy cows under practical feeding conditions. Most mechanistic data originate from in vitro incubations or from non-lactating animals and often rely on a limited number of model compounds such as quercetin, isoflavones or green tea catechins (Bešlo et al., 2022; Cheng et al., 1969; Jayanegara et al., 2012; Simpson et al., 1969; Wein et al., 2016). The extent to which these model systems capture the behaviour of complex feed matrices (e.g., mixed silages, agro-industrial by-products) in the rumen remains uncertain. In addition, there is still no consensus on how inter-individual variability in rumen microbial composition and “metabotypes” translates into differences in systemic exposure to specific metabolites and, ultimately, into milk enrichment (Dadáková et al., 2020; González-Barrio et al., 2012; Makarewicz et al., 2021). These gaps emphasize the need for integrative in vivo studies that combine detailed metabolite profiling with microbial and host factors.

## 5 Milk transfer mechanisms and metabolites of dietary polyphenols

Dietary polyphenols—and more commonly their microbially and host-derived metabolites—can appear in bovine milk after intestinal absorption and systemic circulation, reflecting mammary uptake and secretion. Although concentrations are generally low, this metabolic transfer is reproducible across studies and matrices, and can modulate the small-molecule profile of milk (Agulló et al., 2024; Serra et al., 2021). Mechanistically, transfer likely combines passive diffusion of small lipophilic species with transporter-mediated secretion of conjugated anions; the efflux pump ABCG2/BCRP is upregulated in the lactating bovine mammary gland and is known to export glucuronide/sulphate conjugates across the blood–milk barrier (Mao & Unadkat, 2015; Mahnke et al., 2016).

### 5.1 Evidence of transfer

Some evidences support the notion that polyphenol compounds present in the bovine diet can be transferred into milk, primarily in metabolized forms. Numerous intervention studies have demonstrated that supplementation with polyphenol-rich feedstuffs—including grape pomace, citrus pulp, Moringa oleifera leaf meal, propolis, lemon pulp, red clover, and grape-seed extract—results in detectable increases in either total polyphenols or specific intermediate metabolites of polyphenols in milk (Serra et al., 2021). Targeted metabolomics on commercial cow and goat milks has also catalogued a broad panel of polyphenol conjugates—e.g., phenyl-γ-valerolactone (PVL) sulphates, urolithin glucuronides/sulphates and isoflavone conjugates—supporting the generalisability of diet-to-milk transfer beyond single-trial settings (Agulló et al., 2024). Collectively, these observations indicate that diet can modulate the polyphenol signature of milk, though the extent depends on feed type, compound class, dose rate, basal diet and animal species. Transporter expression (e.g., ABCG2 polymorphisms) may further influence secretion efficiency into milk. (Otero et al., 2015). It is now widely acknowledged that the polyphenol composition of milk is not simply a direct reflection of the ingested compounds, but rather the culmination of a complex cascade of microbial transformations in the rumen, production of conjugates in the intestine, digestion process, host tissue metabolism, and selective mammary uptake. Collectively, these experimental and observational studies show that, even when similar sources or doses of polyphenols are used, the magnitude of milk enrichment and the specific metabolites detected can differ substantially across trials. For example, milk equol and enterolactone concentrations reported in cows and small ruminants supplemented with legume forages or linseed-based diets span a wide range between studies, despite comparable nominal intakes (Brito & Zang, 2018; Ghedini et al., 2017; Křížová et al., 2021). Likewise, investigations using grape by-products or other polyphenol-rich feeds report clear increases in total phenols or antioxidant capacity in some cases, whereas others observe only modest or inconsistent changes in individual metabolites (Bennato et al., 2023; Di Meo et al., 2023; Leparmarai et al., 2019; Orzuna-Orzuna et al., 2023; Serra et al., 2021). This variability likely reflects not only differences in basal diet composition, inclusion level and animal physiology, but also heterogeneity in sampling schemes (e.g., timing relative to feeding, lactation stage) and analytical coverage, which complicates direct comparison of transfer efficiencies across studies.

### 5.2 Specific polyphenol metabolites identified in bovine milk

Progress in analytical methodologies has facilitated the identification and quantification of a wide range of individual polyphenol-derived metabolites in bovine milk, most of which are products of extensive microbial degradation in the rumen and subsequent host metabolic processing. In milk, these species are predominantly phase-II conjugates (glucuronides/sulphates), consistent with systemic handling prior to mammary secretion (Agulló et al., 2024; Mena et al., 2019).

• Isoflavones and Their Metabolites

Among the most frequently studied polyphenols are isoflavones, particularly those derived from soybeans and leguminous forages such as red clover, leading to the generation of biologically active metabolites such as equol and O-desmethylangolensin (O-DMA), which have been consistently detected in bovine milk after appropriate dietary supplementation (Bešlo et al., 2022; Křížová et al., 2021). Equol, due to its potent oestrogenic and antioxidant properties, has been reported at milk concentrations ranging from approximately 4 to 1000 ng/mL, with notably higher values following red clover supplementation compared with soy-based diets (Křížová et al., 2021). In addition to these unconjugated metabolites, commercial milk samples contain conjugated forms—e.g., equol-7-glucuronide, equol-7-sulphate, equol-4′-sulphate and glucuronides of daidzein/dihydrogenistein — with total isoflavone-metabolite concentrations of 141–607 ng/100 mL (Agulló et al., 2024). Isoflavone secretion may also be shaped by mammary transporters; ABCG2 has been implicated in the secretion of polyphenol-like conjugates and related xenobiotics into bovine milk (Mahnke et al., 2016).

• Flavan-3-ol Metabolites

Flavan-3-ols, which include catechins and their polymeric counterparts, proanthocyanidins, are widely present in grape, tea, and cocoa products, and are extensively metabolized by gut microbiota into phenyl-γ-valerolactones (PVLs) and phenylvaleric acids (PVAs) (Mena et al., 2019). Although direct quantification in milk following specific supplementation trials is still limited, commercial cow milk contains several PVLs—mainly as sulphate conjugates (e.g., 5-(hydroxyphenyl)-γ-valerolactone-sulphate and regioisomeric sulphates)—at totals of 161–367 ng/100 mL (Agulló et al., 2024; Mena et al., 2019).

• Ellagitannin Metabolites (Urolithins)

Ellagitannins, derived from dietary sources such as pomegranate, chestnuts, oak leaves, and sainfoin, undergo hydrolysis to yield ellagic acid, which is further metabolized by gut microbiota into urolithins (e.g., A, B, C, D) and their isomers (e.g., isourolithin A) (González-Barrio et al., 2012). These urolithins, detected mainly as glucuronide/sulphate conjugates in circulation, have also been identified in milk (e.g., urolithin A-glucuronide/sulphate, urolithin B-glucuronide/sulphate, urolithin C-glucuronide), with totals of ∼274–514 ng/100 mL in commercial samples (Agulló et al., 2024). The extent of urolithin appearance may vary with the animal ‘metabotype’ (urolithin-A vs. isourolithin-A producers), mirroring inter-individual microbial differences observed in other species (González-Barrio et al., 2012).

• Phenolic Acid Metabolites

A variety of phenolic acid derivatives, arising either from direct ingestion or from the microbial metabolism of more complex polyphenols, are commonly detected in bovine milk. Among these, hippuric acid and 3′-hydroxyhippuric acid occur at relatively high levels (≈ 1211–1872 µg/100 mL in commercial milk) (Agulló et al., 2024). Hippuric acid, a detoxification product of benzoic acid, is considered a general indicator of plant-derived aromatic compound metabolism rather than a specific marker of a single phenolic acid source. Additionally, derivatives of flavonols such as quercetin—including 3,4-dihydroxyphenylacetic acid (3,4-DHPAA)—have been identified in ruminants and reported in human milk (Harun et al., 2025). Moreover, benzene-diol sulphates (putative dihydroxybenzene-sulphate isomers) have been quantified in bovine milk at 4.0–9.7 ng/100 mL (Agulló et al., 2024).

• Tea Catechin Metabolites

Although green tea extract (GTE) and tea polyphenols (TPs) are sometimes used in dairy cow diets for their antioxidant properties, specific quantification of resulting tea catechin metabolites in milk remains sparse. Intraduodenal administration of GTE, bypassing ruminal metabolism, raises plasma epicatechin, epigallocatechin and EGCG (Wein et al., 2016), but consistent, quantitative milk data after in vivo supplementation are still scarce. Where present, catechins and their conjugates would be expected to follow the general pattern of low-abundance, mainly conjugated species in milk (Ma et al., 2016; Wein et al., 2016).

Overall concentration landscape and relevance. It is noteworthy that, aside from hippuric acid, the majority of individual polyphenol metabolites identified in bovine milk are present at low concentrations—generally in the range of a few to several hundred nanograms per 100 mL as summarized above (Agulló et al., 2024; Křížová et al., 2021). While these pools may contribute to antioxidant potential, the physiological significance for human health remains uncertain and likely depends on metabolite identity, conjugation state, potential synergy and habitual intake (Serra et al., 2021).

Variability and remaining uncertainties. When the available studies are considered together, it becomes evident that transfer efficiencies of polyphenols and their metabolites into milk are highly variable and, in many cases, only partially understood. For instance, equol and enterolactone can reach measurable concentrations in milk, but their levels differ markedly across studies and feeding regimes, likely reflecting differences in basal diets, polyphenol intake, gut microbial metabotypes and lactation stage (Brito & Zang, 2018; Ghedini et al., 2017; Křížová et al., 2021). Similarly, data on grape-derived phenolics and their metabolites in milk remain fragmented and sometimes inconsistent, owing to heterogeneous inclusion rates, feed matrices and analytical sensitivity (Bennato et al., 2023; Di Meo et al., 2023; Leparmarai et al., 2019; Rocchetti et al., 2022). In addition, most studies report concentrations of a limited panel of metabolites, without a full mass balance of ingested versus secreted polyphenols, making it difficult to quantify true transfer yields. Altogether, these limitations highlight that our current understanding of feed-to-milk transfer is still incomplete, and that more standardized study designs and broader metabolite coverage are needed to allow robust cross-study comparisons.

Biological relevance and functional implications of milk-borne metabolites. Beyond their value as biomarkers of polyphenol exposure, several classes of metabolites identified in bovine milk are known or hypothesised to possess biological activities that may be relevant for animal health and for the functional properties of dairy products. Isoflavone-derived compounds such as equol and O-desmethylangolensin have been extensively studied for their endocrine-modulating, antioxidant and potential cardiometabolic effects in monogastric models (Gong et al., 2023; Yuan et al., 2007). In dairy cows, equol and enterolactone can be transferred into milk following the ingestion of legume forages or flaxseed, although transfer efficiencies vary widely (Brito & Zang, 2018; Ghedini et al., 2017; Křížová et al., 2021). These lignan and isoflavone metabolites may contribute to the modulation of oxidative status, fertility and inflammatory responses reported in ruminants supplemented with polyphenol-rich feeds (Bešlo et al., 2022; Harun et al., 2025; Serra et al., 2021; ), even though direct causal links are still difficult to demonstrate. Microbial metabolites of hydrolysable tannins, particularly urolithins, have attracted considerable attention due to their anti-inflammatory, antioxidant and mitophagy-promoting activities in non-ruminant species (Espín et al., 2013; Tomás-Barberán et al., 2014). Likewise, colonic metabolites of flavan-3-ols such as phenyl-γ-valerolactones and phenylvaleric acids have been implicated in vascular, metabolic and gut-barrier effects (Mena et al., 2019), while dihydrocaffeic acid and related phenolic acids exhibit strong radical-scavenging and cytoprotective properties (Zieniuk, 2023). Although only low concentrations of these compounds have so far been quantified in bovine milk (Bennato et al., 2023; Rocchetti et al., 2022), they may still exert local effects in the mammary gland or contribute, together with classical lipophilic antioxidants, to the overall antioxidant and anti-inflammatory potential of milk (Di Meo et al., 2023; Huang et al., 2019; Khan et al., 2019). In addition, several intervention studies and reviews indicate that polyphenol-rich diets can improve markers of oxidative and immunometabolic status in dairy animals and modify milk fatty acid profiles, with potential benefits for product stability and nutritional value (Bennato et al., 2023; Harun et al., 2025; Huang et al., 2019; Ma et al., 2021; Ponnampalam et al., 2022; Renna et al., 2022; Xu et al., 2022). However, the relative contribution of specific milk-borne metabolites versus systemic effects of polyphenols on animal physiology remains largely unresolved. These aspects are further discussed in 8, 9, where implications for milk quality and potential human health outcomes are examined.

### 5.3 Determinants of mammary uptake and secretion

Mammary transfer of dietary polyphenols is influenced by: (i) plasma availability (dose rate, matrix (feed type), microbial ‘metabotype’); (ii) protein binding ability and partitioning in the milk matrix (protein–polyphenol interactions); and (iii) transporter activity, notably ABCG2/BCRP, which exports a wide range of glucuronide/sulphate conjugates and is functionally expressed in the bovine mammary epithelium. Genetic variants (e.g., Y581S) can also modify efflux into milk for relevant substrates (Mahnke et al., 2016; Mao & Unadkat, 2015; Otero et al., 2015; Rocchetti et al., 2022).

A synthesis of these findings, detailing key classes of polyphenol metabolites identified in bovine milk along with representative compounds and their concentration ranges, is presented in Table 3.

**Table 3:** Key dietary polyphenols and their metabolites identified in bovine milk following supplementation of polyphenol-rich feeds.

Study / species	Dietary source / precursor polyphenols	Key metabolites detected in milk	Analytical method (sample prep + detection)	References
Dairy cows	Different feeding regimens (grass-, hay-, and concentrate-based diets) providing varying levels of polyphenols and isoflavonoids	Parent polyphenols, isoflavones (e.g., daidzein, genistein), equol, other microbial-derived metabolites	Protein precipitation and extraction followed by UHPLC–QTOF-MS (targeted/untargeted metabolomics)	Rocchetti et al. (2022)
Dairy ewes	Grape pomace supplementation (10 % DM)	Phenolic acids and flavonoids (e.g., luteolin, quercetin derivatives) in milk	Extraction and analysis using UHPLC system with appropriate detection (e.g., UV/DAD and/or MS as described by the authors)	Bennato et al. (2023)
Dairy cows / calves	Legume forages, flaxseed, or enterolactone-enriched milk	Enterolactone, equol and related isoflavone/lignan metabolites	Sample clean-up followed by HPLC or LC–MS/MS quantification of lignans/isoflavones	Ghedini et al. (2017); Brito and Zang (2018); Křížová et al. (2021)
Dairy small ruminants / cows	Other polyphenol-rich by-products (e.g., cocoa bean shell, citrus, tea extracts)	Mixture of parent polyphenols and microbial metabolites (phenolic acids, flavonoid derivatives)	Extraction with LLE/SPE followed by HPLC–DAD or LC–MS/MS, depending on the study	Renna et al. (2022); Di Meo et al. (2023); Huang et al. (2019); Xu et al. (2022)

EGCG = epigallocatechin gallate; EGC = epigallocatechin; PVL = phenyl-γ-valerolactone; DHPAA = dihydroxyphenylacetic acid.

### 5.4 Mechanisms of transfer across the blood-mammary barrier

The transfer of polyphenol metabolites from the bloodstream into milk requires passage across the blood–mammary barrier formed by mammary epithelial cells (MECs). Current evidence indicates contributions from passive diffusion and carrier-mediated transport, analogous to pathways known for nutrients and xenobiotics (Gong et al., 2024; Shivashankara & Acharya, 2010).

**Passive Diffusion:** Smaller, more lipophilic and largely uncharged polyphenol or their conjugates with sufficient membrane permeability may cross MEC membranes down a concentration gradient. Transfer efficiency depends on molecular size, pK_a/ionisation at physiological pH, and lipophilicity (logD), as described for drug partitioning into mammary gland to breast milk (Fiecke et al., 2025; Gong et al., 2024; Nguyen & Neville, 1998).

**Carrier-Mediated Transport:** It is highly likely that membrane transporters on the basolateral (blood-facing) and apical (milk-facing) MEC membranes facilitate uptake and secretion of certain polyphenol conjugates. ABCG2/BCRP—robustly induced during lactation—actively secretes diverse substrates into milk, classically including riboflavin; ABCG2 knockout in mice reduces milk riboflavin >60-fold (van Herwaarden et al., 2007). In cattle, the gain-of-function ABCG2 polymorphism Y581S increases secretion of known substrates into milk, underscoring transporter control of milk efflux; variants also affect secretion of natural compounds such as enterolactone (Otero et al., 2015, 2016). Beyond ABCG2, solute-carrier (SLC) transporters likely contribute to basolateral uptake and apical efflux of conjugated anions (e.g., glucuronides/sulphates), as suggested above by xenobiotic and nutrient transport paradigms in the mammary gland (Gong et al., 2024).

**Barrier integrity and paracellular leak:** Tight junctions between MECs normally restrict paracellular movement of small solutes; their closure is a hallmark of established lactation. During mastitis, milk stasis or early/late lactation transitions, junctions become more permeable, potentially increasing non-specific transfer between blood and milk across the mammary gland cells (Nguyen & Neville, 1998; Stelwagen & Singh, 2014; Wellnitz & Bruckmaier, 2021).

**Binding to Milk Components:** Once in milk, polyphenols can interact with proteins and colloidal structures, altering apparent concentrations and stability. Tea catechins bind β-lactoglobulin and associate with casein micelles; protein–polyphenol complexes can protect polyphenols against oxidation and modulate bioaccessibility (Khan et al., 2019; van de Langerijt et al., 2023). Although many such studies derive from in vitro experiments or direct milk fortification, if these interactions occur post-transfer in vivo, they could significantly affect the stability, solubility, and measurable concentrations of these compounds within the milk matrix. Additionally, casein micelles have demonstrated the capacity to associate with polyphenols, which may contribute to their sequestration or stabilization in milk (Khan et al., 2019). Processing and matrix factors (pH, heat, carbohydrate interactions) further shape protein–polyphenol binding and recovery in analytical workflows (Wijegunawardhana et al., 2024).

**MEC cellular responses and local metabolism:** MECs are not passive conduits: *ex vivo* exposure of caprine MECs to plasma from goats consuming phenolic-rich lentisk increased intracellular casein and triglyceride, and enhanced ATP production/oxygen consumption (Hadaya et al., 2021). In vitro, tea polyphenols increased antioxidant capacity and down-regulated pro-inflammatory signalling in bovine MECs, indicating direct cytoprotective actions that could influence secretion dynamics under stress (Ma et al., 2022; Xu et al., 2022).

**Mechanistic summary:** Overall, mammary transfer of dietary polyphenols reflects: (i) systemic availability of conjugated/parent forms; (ii) transporter-mediated efflux (notably ABCG2, with genetic and physiological modulation); (iii) tight-junction status (paracellular leak during inflammation or transition states); and (iv) post-transfer binding within the milk matrix that can stabilise or sequester polyphenols and influence their measured levels.

## 6 Factors influencing polyphenol bioavailability and their transfer to milk

The journey of dietary polyphenols from feed to milk is governed by a complex interplay of factors related to the chemical nature of the polyphenols themselves, the composition of the diet matrix, and physiological and microbial characteristics of the animal. A comprehensive understanding of these factors is essential to develop effective strategies aimed at enriching milk with specific bioactive compounds. The concept of “bioavailability” in ruminants is particularly complex due to extensive ruminal microbiology, which often transforms parent polyphenols into microbial metabolites and further as conjugates in the intestine more likely to become systemically available and subsequently transferred into milk. Therefore, factors influencing rumen function are of paramount importance in the transformation of dietary polyphenols into milk compounds.

### 6.1 Polyphenol-related factors

The inherent chemical structure and class of a polyphenol primarily determine its metabolic fate within the ruminant system. Bioavailability and metabolic pathways vary considerably among different polyphenol classes and individual compounds (Chen et al., 2018; Teng & Chen, 2019). For example, in the small intestine, polyphenol aglycones are generally absorbed more readily than their corresponding glycosides, although ruminal hydrolysis frequently releases aglycones, making them available for further microbial metabolism or direct absorption from the rumen (Chen et al., 2018). In addition to glycoside presence, structural parameters such as molecular size, polarity and degree of polymerization have a profound impact on absorption efficiency and downstream metabolism (Lewandowska et al., 2013). Isoflavones, for instance, tend to exhibit higher bioavailability compared to more complex polyphenols such as anthocyanins or highly polymerized proanthocyanidins (Hendrich, 2002). The dosage or intake level similarly influences the concentration of metabolites in plasma and, by extension, their potential transfer into milk (Leparmarai et al., 2019). Some dose-dependent effects have been reported; supplementation with grape seed proanthocyanidin extract (GSPE) at 20 mg/kg body weight increased milk yield in dairy cows, whereas higher doses (40 and 80 mg/kg BW) did not produce further increases, and the effects on milk protein and non-fat solids were transient (Huang et al., 2019). In small ruminants, supplementation with grape-seed extract increased total polyphenols in milk, showing a weak positive correlation between polyphenol intake and milk polyphenol secretion in sheep (Leparmarai et al., 2019). Non-linear (often quadratic) responses are common with plant bioactives, reflecting threshold effects, microbial adaptation and potential saturation of transport/conjugation pathways (Harun et al., 2025). Moreover, chemical stability under gastrointestinal conditions is critical. Many polyphenols are unstable at ruminal pH or undergo extensive microbial degradation into metabolites (e.g., phenolic acids) that may possess greater systemic bioactivity than their parents (Selma et al., 2009; Tomás-Barberán et al., 2014). For example, quercetin is rapidly degraded in the rumen to 3,4-dihydroxyphenylacetic acid, detected in plasma and milk following supplementation (Berger et al., 2015). Thus, considering both parent compounds and their microbial derivatives is essential when evaluating bioavailability and transfer potential. In some cases, enterohepatic recirculation may prolong systemic presence of polyphenol metabolites, enhancing the probability of secretion into milk (Bešlo et al., 2022). Binding to plasma proteins (e.g., albumin) also modulates distribution and mammary availability (Dangles et al., 2001). Kinetic and metabolomic time-course studies—tracking free and conjugated forms—remain limited in ruminants and are needed to define exposure–response windows.

### 6.2 Diet-related factors (feed matrix)

The overall composition of the diet (feed matrix) significantly affects bio-accessibility, uptake and metabolism of polyphenols (Bohn, 2014). Dietary fibre (e.g., hemicellulose), divalent minerals and protein-rich meals can reduce bio-accessibility by binding or sequestering polyphenols (Bohn, 2014). Conversely, digestible carbohydrates and dietary lipids may enhance the availability of more hydrophobic compounds (Bohn, 2014). Notably, milk co-ingestion with tea has been shown in vitro to stabilise green-tea catechins and potentially enhance their transport across intestinal models (Xie et al., 2013). Certain forages, particularly red clover, contain high levels of polyphenol oxidase (PPO). PPO is activated upon tissue damage during harvesting, ensiling or mastication, oxidizing endogenous o-diphenols to o-quinones that covalently bind proteins and other nucleophiles, forming protein-bound polyphenols (PBP) and related complexes (Lee, 2014). PBP formation improves nitrogen-use efficiency and can modify the milk fatty-acid profile by protecting dietary proteins and PUFA from degradation during silage fermentation and ruminal digestion (Lee, 2014). The extent to which PPO activity or PBP formation modulates the absorption/transfer of non-substrate polyphenols remains unclear and likely context-dependent, warranting targeted studies.

Moreover, silage-rich diets influence the redox state and enzymatic environment of the rumen, potentially altering degradation kinetics and the spectrum of microbial metabolites (Tamminga et al., 1994). Silage fermentation may also generate novel polyphenol fermentation products with distinct absorption profiles (De Bellis et al., 2022; Formato et al., 2022). Particle size and physical structure modulate release during digestion. Finely ground feeds can increase surface area and enhance enzymatic access, improving liberation from the matrix. In contrast, coarser diets might prolong rumen retention time, favoring more extensive microbial transformation (Maggiolino et al., 2025). Thus, feed form and physical processing should be considered alongside chemical composition when evaluating polyphenol availability. Another consideration is the presence of emulsifiers or surfactants, whether naturally occurring (e.g., bile acids) or added as feed additives. These compounds can enhance solubilization of hydrophobic polyphenols, potentially improving their micellar incorporation and intestinal uptake (Kaur & Kaur, 2014; Sazdanić et al., 2023). While such strategies are commonly studied in monogastric animals, their application in ruminant systems remains largely unexplored but potentially promising and warrant investigation of well-designed in vivo studies.

### 6.3 Interaction effects and synergistic influences

Although factors have been discussed independently, the interactions among them may exert synergistic or antagonistic effects on polyphenol bioavailability. For instance, certain dietary polyphenols can modulate the gut or rumen microbiota in ways that feedback on their own metabolism, leading to either enhanced or suppressed metabolite formation (Orzuna-Orzuna et al., 2023; Teng et al., 2024). Moreover, the simultaneous presence of multiple polyphenol classes in the diet may result in competitive inhibition or mutual stabilization, affecting microbial degradation patterns and subsequent absorption (Hassan et al., 2020; Wanapat et al., 2024). Some polyphenols, such as tannins, can bind to both dietary proteins and other polyphenols, potentially limiting the systemic availability of both (Besharati et al., 2022; Patra & Saxena, 2011). Factorial designs and response-surface approaches are recommended to resolve non-linear, matrix-dependent effects in vivo. These interaction effects are often nonlinear and context-dependent, underlining the need for factorial experimental designs in future research. Additionally, co-supplementation of polyphenols with other dietary bioactives, such as essential oils, vitamins, or minerals, may yield synergistic effects on ruminal fermentation and microbial activity (Choi & Kim, 2020). For example, vitamin E and selenium have been shown to interact with flavonoids in modulating oxidative stress and immune response, potentially affecting systemic distribution and milk secretion of polyphenol-derived compounds (Andrés et al., 2024). Inclusion of probiotics or prebiotics in the diet can also influence the gut microbial landscape, which in turn affects the metabolic fate of polyphenols. Certain lactic acid bacteria are capable of hydrolyzing polyphenol glycosides or converting polyphenols into bioactive phenolic acids, thus potentially increasing their bioavailability and systemic concentrations (Ahmed et al., 2024). The extent to which these microbial interactions occur in ruminants remains to be fully characterized, but initial studies suggest that manipulating the microbial consortia could optimize the bioconversion of dietary polyphenols into metabolites with improved transferability into milk. Moreover, the physical and chemical properties of the gastrointestinal environment — such as rumen pH, redox potential, and transit time — are themselves influenced by diet composition and feeding behavior. These factors not only modulate microbial enzymatic activity but also affect polyphenol stability and solubility, introducing another layer of interaction (Bohn, 2014). Such indirect and cascading interactions make it challenging to predict polyphenol bioavailability from single-factor analyses alone.

### 6.4 Animal-related factors

The rumen microbial ecosystem is the primary determinant of polyphenol metabolism in dairy cows. The specific composition and activity of bacteria, protozoa, and fungi in the rumen can influence the metabolic pathways and extent of transformation of ingested polyphenols (Makarewicz et al., 2021). Significant inter-individual variation in rumen microbiota composition leads to notable differences in polyphenol metabolite profiles between animals fed identical diets (González-Barrio et al., 2012). Beyond microbial factors, host genetics also affect metabolism, absorption, and secretion of polyphenols or their metabolites into milk. The concept of “metabotypes” — classifying individuals based on their ability to produce specific metabolites such as equol from isoflavones — is well established in human nutrition (D’Archivio et al., 2010) and likely applies to dairy cows, contributing to variability in milk polyphenol profiles. Recent studies suggest that the presence of certain microbial strains (e.g., *Slackia isoflavoniconvertens, Eggerthella* spp., and *Lactobacillus* spp.) may confer higher efficiency in converting polyphenols into bioactive metabolites such as equol or urolithins, which have greater systemic bioactivity and milk transfer potential (Makarewicz et al., 2021). Thus, strategies aimed at modulating or stabilizing microbial communities—through probiotic supplementation, fecal transplants, or selective breeding—may enhance polyphenol metabolite consistency and bioavailability across herds.

Physiological status, including lactation stage and parity, influences overall milk composition and metabolite transfer. For example, total protein and whey protein concentrations typically increase as lactation progresses (Nudda et al., 2003), and metabolic signatures vary between early and late lactation milk (Connolly et al., 2023). Although these factors affect milk chemistry broadly, their specific impact on concentrations of polyphenol metabolites remains poorly understood. Seasonal variations in forage availability (e.g., red clover content) also affect milk polyphenol content, highlighting the interconnectedness of diet, physiology, and metabolite transfer (Johansen et al., 2018; Moorby et al., 2009). Mammary gland health and somatic cell count (SCC) further influence milk composition. Elevated SCC due to mastitis or subclinical inflammation alters milk fat, lactose, and protein fractions (Nudda et al., 2003), and impaired blood-milk barrier integrity may affect metabolite passage into milk (Zhang et al., 2025). Interestingly, some polyphenol supplements, such as flavonoids, have been reported to reduce SCC, suggesting beneficial effects on udder health that could indirectly enhance metabolite transfer (Orzuna-Orzuna et al., 2023). Emerging evidence also highlights the role of hormonal and immunological responses in modulating transporter expression in the mammary epithelium. For instance, inflammatory cytokines may downregulate ABC transporters (e.g., BCRP/ABCG2), which are implicated in the active secretion of certain polyphenol metabolites into milk (García-Lino et al., 2019). Thus, systemic health status not only affects rumen and intestinal metabolism but may also regulate milk secretion pathways.

Finally, the efficiency of intestinal absorption and milk secretion systems modulate systemic availability of polyphenols and their metabolites. Enzymes such as lactase phloridzin hydrolase (LPH) and cytosolic β-glucosidase (CBG) facilitate hydrolysis of glycosides in the small intestine (D’Archivio et al., 2010), while efflux transporters like P-glycoprotein can limit absorption by pumping compounds back into the intestinal lumen (Bohn, 2014). The balance of these processes ultimately determines the concentration of bioactive compounds available for secretion into milk. In summary, animal-related factors encompass a wide range of biological processes, from rumen microbial dynamics and intestinal enzymatic activity to host genetics, health status, and mammary gland physiology. In addition to the fundamental research, future research should integrate omics-based tools (metagenomics, transcriptomics, metabolomics) to holistically capture these interdependent influences on polyphenol metabolism and milk enrichment in ruminants.

### 6.5 Influence of feed processing and storage

Processing methods such as pelleting, extrusion, and ensiling significantly affect the stability and release of polyphenols in feed. Thermal processing can degrade heat-sensitive compounds, while ensiling may enhance or reduce polyphenol extractability depending on fermentation dynamics and PPO activity (Chaaban et al., 2017; De Bellis et al., 2022; Oancea, 2021). The effect of pelleting and extrusion on polyphenols is highly dependent on temperature, moisture, and residence time. While moderate heat may facilitate the release of bound polyphenols through cell wall disruption, excessive thermal treatment can lead to structural degradation or oxidation of polyphenol moieties, particularly in flavonoid-rich feeds (Arranz et al., 2010). Some evidence also suggests that pelleting may reduce anti-nutritional tannin activity in legume forages, indirectly improving nutrient absorption and possibly enhancing systemic polyphenol bioavailability (Oyaniran et al., 2018). Storage conditions (temperature, light, oxygen) also influence the integrity of polyphenol-rich feed ingredients and should be considered when assessing effective intake levels in vivo. Polyphenols such as catechins and anthocyanins are particularly prone to degradation via photo-oxidation and auto-oxidation during prolonged storage (Ignat et al., 2011). The use of oxygen-barrier silage films or vacuum-packaging techniques has been proposed as a way to reduce oxidative losses during storage, especially in high-value functional forages like red clover or grape pomace silage (Formato et al., 2022).

In addition, silage fermentation may generate novel bioactive compounds, whose metabolic fate in the ruminant remains largely uncharacterized (Formato et al., 2022). These fermentation-derived compounds include low-molecular-weight phenolic acids and microbial metabolites that can either retain bioactivity or act as precursors to more active forms after ruminal or intestinal transformation Moreover, the inoculation of silage with specific microbial starters (e.g., *Lactobacillus plantarum* or *Lactobacillus buchneri*) has been shown to modulate polyphenol profiles during ensiling, potentially enriching the feed with metabolites such as ferulic acid, coumaric acid, or bioavailable flavonoid glycosides (Yan et al., 2025). Nevertheless, variability in fermentation conditions—such as pH, water activity, and microbial contamination—introduces considerable heterogeneity in polyphenol retention and transformation across silage batches. Thus, standardized silage management and real-time monitoring of fermentation dynamics could help stabilize polyphenol delivery and improve reproducibility of

### 6.6 The bioavailability–bioactivity paradox in dairy cows and their milk

A recurring theme in the polyphenol literature is the so-called “bioavailability–bioactivity paradox”, whereby relatively low systemic concentrations of parent compounds and metabolites coexist with measurable biological effects in vivo (Bohn, 2014; D’Archivio et al., 2010; Teng & Chen, 2019). This paradox is also apparent in dairy cows. In vivo kinetic studies with model compounds such as catechins and ellagitannin-derived metabolites suggest that only a small fraction of the ingested dose appears in plasma as identifiable entities, and that the proportion recovered in milk is even lower (González-Barrio et al., 2012; Wein et al., 2016). Consistently, targeted and untargeted metabolomics analyses usually detect polyphenol-derived metabolites in bovine milk at low micromolar or sub-micromolar concentrations, often close to the limits of quantification (Agulló et al., 2024; Bennato et al., 2023; Rocchetti et al., 2022). In apparent contrast with these low concentrations, multiple intervention studies and reviews report improvements in antioxidant and immunometabolic status, modulation of rumen fermentation and methane emissions, and favourable changes in milk composition in response to polyphenol-rich feeds or phytogenic additives (Ahmed et al., 2024; Formato et al., 2022; Harun et al., 2025; Huang et al., 2019; Ma et al., 2021; Ponnampalam et al., 2022; Serra et al., 2021; Xu et al., 2022). Several, non-mutually exclusive mechanisms may contribute to reconciling these findings. First, many microbial and phase II metabolites, including urolithins, phenyl-γ-valerolactones, phenylvaleric acids and dihydrocaffeic acid, exhibit significant bioactivity in cell and animal models at low concentrations, often through modulation of signalling pathways rather than direct radical scavenging (Espín et al., 2013; Mena et al., 2019; Zieniuk, 2023). Second, local effects within the gastrointestinal tract and the mammary gland, mediated by microbiota–host interactions, barrier function and immune cell modulation, may be more important than systemic antioxidant effects per se (Makarewicz et al., 2021; Santhiravel et al., 2022; Selma et al., 2009; Xu et al., 2022). Third, repeated dietary exposure over days to weeks can lead to sustained modulation of gene expression, enzyme activities and microbial community structure, so that functional outcomes may depend more on cumulative signalling than on peak plasma levels (Lewandowska et al., 2013; Manach et al., 2004; Teng & Chen, 2019). From the perspective of milk as a functional food, this paradox implies that the health-relevant value of “polyphenol-enriched” milk cannot be judged solely on the basis of total polyphenol content or a few marker metabolites. Instead, both the spectrum of bioactive microbial- and host-derived metabolites delivered to the consumer and the upstream effects of dietary polyphenols on animal health, milk composition and technological properties need to be considered. Taken together, these compound-, diet-, animal- and processing-related factors clearly interact in a highly non-linear manner. However, most published studies manipulate one factor at a time (e.g., dose of a single extract, inclusion of one by-product), making it difficult to disentangle main effects from interactions and to derive predictive relationships for milk enrichment (Herremans et al., 2020; Johansen et al., 2018; Ponnampalam et al., 2022). Multifactorial experimental designs and modelling approaches explicitly integrating dietary composition, rumen function and mammary physiology are still scarce. This represents an important gap, particularly for the development of precision feeding strategies that aim to achieve reproducible polyphenol delivery to the mammary gland.

## 7 Analytical advances for quantifying polyphenols and their metabolites in milk

The accurate identification and quantification of polyphenols and their metabolites in milk pose significant analytical challenges, largely due to the inherent complexity of both the analytes and the milk matrix. Substantial progress in plant-matrix analytics does not directly translate to milk, where protein–lipid colloids and conjugated, low-abundance metabolites demand tailored workflows from sampling to data analysis (Chiriac et al., 2021; Hu et al., 2022; Iqbal et al., 2022; Ivanović et al., 2020; Sun et al., 2022; Usman et al., 2022; Zhang et al., 2022).

### 7.1 Challenges in analysis

Several factors contribute to the difficulty of analyzing polyphenols in milk. Firstly, polyphenols and their metabolites are typically present at very low concentrations, often in the nanogram per milliliter (ng/mL) range or lower, necessitating highly sensitive detection methods (Daems et al., 2016). Secondly, milk is a complex biological fluid rich in fats, proteins, lactose and minerals, all of which can interfere with extraction, separation and detection. Matrix effects (ion suppression/enhancement) are common in LC–MS and must be evaluated and controlled (Daems et al., 2016; Matuszewski et al., 2003). Additionally, the chemical diversity of polyphenols is extensive—parent forms (aglycones/glycosides), phase-II conjugates (glucuronides, sulphates, methylated derivatives) and microbial products—so methods must accommodate a broad polarity/chemistry window (González-Barrio et al., 2012). Another considerable obstacle is the limited availability of commercial analytical standards, particularly for microbial metabolites and conjugates, complicating unequivocal identification and accurate quantification; surrogate standards and semi-quantification are often used (González-Barrio et al., 2012). Finally, lack of standardised protocols for sample preparation and analysis (e.g., for isoflavones) hampers inter-study comparability (Daems et al., 2016). Practical QA/QC issues include analyte instability (pH, light, temperature, oxidation), adsorption to plastics, and carryover; these necessitate stabilisers (e.g., ascorbate/EDTA), amber vials, chilled autosamplers and rigorous blanks (Daems et al., 2016; Ignat et al., 2011).

### 7.2 Sample preparation

Effective sample preparation represents a critical preliminary step to isolate polyphenols from the milk matrix, remove interfering substances, and concentrate analytes prior to instrumental analysis. Because polyphenols bind to milk proteins (caseins, whey) via H-bonding/hydrophobic interactions, pretreatment must address both proteins and lipids (Helal et al., 2014; Yildirim-Elikoglu & Erdem, 2018)

**Protein removal.** Acid precipitation can denature proteins but may compromise polyphenol integrity/recovery; it should be used cautiously (Miniati, 2007). Organic-solvent deproteinisation is widely adopted for milk due to simplicity and performance: acetonitrile > acetone > ethanol for efficacy (Chiavelli et al., 2020). Centrifugation then allows the removal of both precipitated proteins pellet and fat (Daems et al., 2016).

**Extraction/clean-up.** Liquid–liquid extraction (LLE) with ethyl acetate (often after mild acidification) and solid-phase extraction (SPE) with C18 or polymeric sorbents are common (Daems et al., 2016). DLLME (dispersive LLE) miniaturises LLE and can enhance recovery of diverse phenolics (e.g., gallic acid, catechins, isoflavones) from human/animal milk. Phospholipid-removal or mixed-mode cartridges help reduce ion suppression in LC–MS (Daems et al., 2016). Solid-phase extraction (SPE) is widely employed for sample clean-up and concentration, with sorbent materials chosen according to the polarity of the target analytes (e.g., C18, polymeric phases). In the case of milk, whose high fat and protein content generates strong matrix effects, SPE is particularly useful for reducing interferences and enriching polyphenols (Daems et al., 2016).

**Conjugates vs aglycones.** Since many polyphenol metabolites occur as glucuronide or sulfate conjugates in milk, hydrolysis is frequently performed to quantify total aglycone content or to simplify chromatographic profiles. Enzymatic hydrolysis, often employing crude enzyme preparations from Helix pomatia containing β-glucuronidase and sulfatase activities, is the most common approach. This method cleaves both glucuronide and sulfate conjugates, releasing free aglycones. Challenges with enzymatic hydrolysis include ensuring reaction completeness, possible enzyme inhibition by matrix components, and the risk of endogenous polyphenols in enzyme preparations interfering with analysis (Daems et al., 2016; González-Barrio et al., 2012). Enzyme blanks and recovery checks are essential due to potential endogenous polyphenols in crude enzyme preps and incomplete hydrolysis; parallel “intact-conjugate” analyses are recommended to avoid over-estimating aglycones. Acid hydrolysis may also be used but can be too harsh for labile polyphenols, potentially causing degradation (Daems et al., 2016).

For certain polyphenols, particularly less volatile or thermally labile phenolic acids analyzed by gas chromatography (GC), derivatization is required. Sialylation, using reagents such as BSTFA or TMCS, replaces active hydrogens with trimethylsilyl groups, thereby increasing volatility and thermal stability, rendering the compounds suitable for GC analysis.

Meticulous execution of sample preparation is paramount; even the most advanced analytical instrumentation cannot yield reliable data if analytes are not efficiently extracted, purified from interferences, and adequately concentrated.

### 7.3 Separation techniques

Liquid chromatography (LC) is the principal tool. RP-HPLC/UHPLC on C18 phases separates moderately polar to non-polar analytes; UHPLC improves speed, resolution and sensitivity using sub-2-µm particles and acidified aqueous–organic gradients (González-Barrio et al., 2012). Ultra-high-performance liquid chromatography (UHPLC) has been essential in resolving complex polyphenol metabolite mixtures, often utilizing binary or ternary mobile phase gradients composed of acidified water (e.g., with formic or acetic acid to suppress phenolic acid ionization and improve peak shape) and organic solvents such as acetonitrile or methanol (Daems et al., 2016). On the contrary, gas chromatography (GC) is less used as most polyphenols are non-volatile/thermally labile; it remains useful for selected volatile phenolic acids after derivatization. For highly polar conjugates (glucuronides/sulphates), complementary selectivity from HILIC or mixed-mode (anion-exchange/reversed-phase) can improve retention and separation, aiding isomer resolution prior to MS (Daems et al., 2016).

### 7.4 Detection techniques

Detection methods must be both sensitive and selective to accurately identify and quantify the typically low polyphenol concentrations in milk. Ultraviolet-visible (UV/Vis) spectroscopy coupled with diode array detection (DAD or PDA) is commonly paired with LC systems and measures absorbance based on the characteristic UV spectra of aromatic polyphenols. While this method allows acquisition of full spectra for peak identification and purity assessment, its sensitivity may be insufficient for low-level metabolites, and certain compounds such as equol have poor UV absorbance (Daems et al., 2016; González-Barrio et al., 2012).

Mass spectrometry (MS) has emerged as the gold standard due to its superior sensitivity, selectivity, and capability to provide structural information. MS is generally coupled to LC or UHPLC systems (LC-MS, LC-MS/MS, UHPLC-MS/MS). Electrospray ionization (ESI), often operated in negative ion mode to favor polyphenol deprotonation, is the most frequently used ionization technique; atmospheric pressure chemical ionization (APCI) is also applied for less polar compounds (Daems et al., 2016).

Mass analyzers vary in their applications: triple quadrupole (QqQ) MS excels in targeted quantitative analysis through selected ion monitoring (SIM) or multiple reaction monitoring (MRM), providing high selectivity and sensitivity (González-Barrio et al., 2012). Time-of-flight (TOF) and quadrupole time-of-flight (QTOF) MS offer high mass accuracy and resolution, facilitating identification of unknown compounds by elemental composition and confirming known metabolites; QTOF instruments also support MS/MS fragmentation for structural elucidation (González-Barrio et al., 2012). Ion trap (IT) MS can perform multiple fragmentation stages (MSⁿ), providing detailed structural data to assist in identifying complex polyphenol metabolites (González-Barrio et al., 2011). High-resolution PRM (Orbitrap/Q-TOF) and data-independent acquisition (DIA, e.g., SWATH) enhance specificity and discovery in complex milk matrices; ion mobility (LC–IMS–MS) can further separate isomers and reduce interferences (Agulló et al., 2024; Daems et al., 2016). Nuclear magnetic resonance (NMR) spectroscopy, while invaluable for unequivocal structural elucidation of novel compounds, is generally less suitable for routine quantification in complex matrices such as milk due to its inherently lower sensitivity compared to MS (Al-Shabib et al., 2020).

Advances in analytical capabilities, particularly the coupling of UHPLC with tandem MS (MS/MS), have increasingly unveiled the complex and detailed profiles of polyphenol metabolites in milk. Many conjugates and microbial degradation products previously undetected or unquantifiable are now accessible, necessitating a shift from targeted quantification of a few parent compounds to more comprehensive metabolomic approaches capable of capturing the true diversity of these bioactive molecules (Agulló et al., 2024).

### 7.5 Quantification approaches

Accurate quantification is essential to assess the extent of polyphenol transfer into milk and to evaluate the functional impact of enriched dairy products. The use of certified authentic reference standards for each analyte remains the most reliable method for generating calibration curves and quantification (Daems et al., 2016). However, due to the scarcity of commercial standards for many polyphenol metabolites, particularly conjugated and microbial derivatives, indirect quantification methods are often employed. These may involve assuming similar detector response factors for metabolites relative to structurally related standards or determining total aglycone content following enzymatic or chemical hydrolysis of conjugates. While practical, these approaches introduce uncertainties (González-Barrio et al., 2012). Internal standards (IS) should be added early to correct extraction and instrument variability. Stable-isotope-labelled IS are preferred; where unavailable, matrix-matched calibration, standard addition and post-extraction spikes are recommended (Agulló et al., 2024; Matuszewski et al., 2003). Reporting should include recoveries, matrix effect (%ME), process efficiency, LOD/LOQ and precision/accuracy from spiked milk. A summary of common analytical techniques is presented in Table 4.

**Table 4:** Common analytical workflows for the quantification of polyphenols and their metabolites in milk. Summary of typical sample preparation, chromatographic and detection techniques used to analyse polyphenols and their metabolites in milk, including main advantages, limitations and typical analytes targeted.

Analytical Step	Technique/Method	Key Advantages	Key Challenges/Limitations	Typical Analytes Targeted	References
Sample Preparation	Liquid-Liquid Extraction (LLE)	Good recovery for many polyphenols, relatively simple.	Solvent selection critical, can be labor-intensive, potential for emulsions with milk.	Broad range of polyphenols.	(Daems et al., 2016).
	Dispersive LLE (DLLME)	Miniaturised; fast; low solvent	Optimisation needed; salt effects	Diverse phenolics (gallic acid, catechins, isoflavones)	(Mohebi et al., 2020)
	Solid-Phase Extraction (SPE)	Effective clean-up, concentration of analytes, can be automated.	Sorbent selection critical, potential for analyte loss if conditions not optimized, cost of cartridges.	Broad range, targeted clean-up based on sorbent.	(Daems et al., 2016).
	Protein precipitation (ACN/acetone/EtOH)	Efficient deproteinisation; simple	Possible co-precipitation of analytes	Most milk workflows	(Chiavelli et al., 2020)
	Enzymatic Hydrolysis (β-glucuronidase/sulfatase)	Cleaves conjugates to allow total aglycone quantification, simplifies chromatograms.	Enzyme purity (contaminants), incomplete hydrolysis, cost, time-consuming incubation.	Glucuronide and sulfate conjugates of flavonoids, phenolic acids, urolithins, etc.	(González-Barrio et al., 2012).
	Defatting/Deproteinization	Removes major matrix interferences (lipids, proteins).	Potential co-precipitation or loss of analytes.	Essential pre-step for most milk analyses.	
Separation	Reverse-Phase HPLC (RP-HPLC)	Robust, widely available, good for moderately polar compounds.	Longer analysis times, lower resolution than UHPLC.	Flavonoids, phenolic acids, tannins, and their metabolites.	(González-Barrio et al., 2012).
	Ultra-High-Performance LC (UHPLC)	Faster analysis, higher resolution, increased sensitivity.	Higher backpressures, requires specialized instrumentation.	Complex mixtures of polyphenol metabolites, isomers.	(Daems et al., 2016)
	HILIC / mixed-mode	Retains very polar conjugates	Method development required	Glucuronides/sulphates	(Daems et al., 2016)
	Gas Chromatography (GC)	Good for volatile compounds.	Requires derivatization for most polyphenols, not suitable for non-volatile or thermally labile compounds.	Volatile phenolic acids (after derivatization).	(Stalikas, 2007)
Detection	UV–Visible / Diode Array Detection (DAD/PDA)	Relatively inexpensive, provides spectral information for identification.	Lower sensitivity than MS, not suitable for all compounds (e.g., poor UV chromophores like equol).	Compounds with strong UV absorbance (most flavonoids, phenolic acids).	(González-Barrio et al., 2012).
	Mass Spectrometry (MS) - Triple Quadrupole (QqQ)	High sensitivity and selectivity for targeted quantification (MRM).	Requires prior knowledge of analytes and transitions, less suited for untargeted screening.	Known polyphenol metabolites and their conjugates.	(González-Barrio et al., 2012).
	Mass Spectrometry (MS) - TOF, QTOF	High mass accuracy for identification of unknowns, good for untargeted metabolomics, structural confirmation.	More complex data analysis, generally higher cost.	Broad screening of known and unknown metabolites, structural elucidation.	(González-Barrio et al., 2012).
	Mass Spectrometry (MS) - Ion Trap (IT)	MSn capabilities for detailed structural elucidation.	Lower resolution and mass accuracy than TOF/QTOF.	Structural characterization of complex polyphenols and metabolites.	(González-Barrio et al., 2011)
	PRM / DIA (SWATH)	Specific/quantitative in complex matrices	Advanced setup/processing	Targeted & broad-scope	(Daems et al., 2016; Agulló et al., 2024)
	LC–IMS–MS	Orthogonal separation of isomers	Instrument access; method dev.	Isomeric conjugates	(Agulló et al., 2024)
Strategy	CIL LC–MS	Enhanced coverage/quant. for phenols	Labelling workflow	Phenol/amine submetabolome	(Mung & Li, 2017)
Quantification	Matrix-matched calibration / standard addition	Compensates matrix effects	More labour	Complex milk matrices	(Matuszewski et al., 2003)

LLE = liquid–liquid extraction; DLLME = dispersive liquid–liquid microextraction; SPE = solid-phase extraction; HPLC = high-performance liquid chromatography; RP-HPLC = reversed-phase HPLC; LC–MS/MS = liquid chromatography–tandem mass spectrometry; PRM = parallel reaction monitoring; DIA = data-independent acquisition; SWATH = sequential window acquisition of all theoretical fragment-ion spectra.

### 7.6 QA/QC and reporting standards

Adopt routine blanks (procedural/instrument), duplicate extractions, spike-recovery at ≥2 levels, and post-column infusion to map ion suppression. Use enzyme blanks for hydrolysis steps and stability checks (freeze–thaw, bench-top). Inter-laboratory comparability benefits from shared reference materials and harmonised SOPs (Daems et al., 2016; Matuszewski et al., 2003). From a comparative perspective, differences in sample preparation, chromatographic separation and MS detection across laboratories are likely to be a major source of discrepancy in which metabolites are reported as present or absent in milk and at what concentrations. Studies focusing on a narrow set of targeted analytes, often based on standards readily available for isoflavones or phenolic acids, inherently overlook other relevant microbial-derived metabolites such as urolithins or phenyl-γ-valerolactones (Espín et al., 2013; Mena et al., 2019; Rocchetti et al., 2022). Conversely, untargeted LC–MS workflows and advanced data-processing pipelines can reveal a much broader spectrum of polyphenols-derived features, but at the cost of more complex identification and variable reporting standards (Chiriac et al., 2021; Mung & Li, 2017; Sun et al., 2022). Furthermore, matrix effects and incomplete recovery remain non-negligible even in carefully optimised methods (Chiavelli et al., 2020; Matuszewski et al., 2003). As a result, cross-study comparisons of absolute concentrations and even of the qualitative metabolite profiles must be made with caution, and there is a clear need for harmonized panels of reference compounds, inter-laboratory comparisons and minimum reporting standards in this field.

## 8 Implications of dietary polyphenols for milk quality and dairy products

The inclusion of polyphenols in the diets of dairy cattle can induce modifications in milk that extend beyond merely increasing its polyphenols metabolite content. These bioactive compounds have been shown to affect multiple facets of milk quality, including antioxidant capacity, fatty acid composition, protein and lactose concentrations, somatic cell count, and even the sensory attributes of dairy products derived. Such changes can significantly influence nutritional value, processing characteristics, and consumer acceptance of milk.

### 8.1 Antioxidant capacity of milk

One of the most consistently documented effects of dietary polyphenols supplementation is the enhancement of the total antioxidant capacity (TAC) of milk (Serra et al., 2021). This improvement has been demonstrated using a variety of polyphenols-rich sources, including Moringa oleifera, pomegranate peel extract, fresh lemon pulp, citrus pulp, grape pomace, olive leaves, Acacia farnesiana extracts, hesperidin, naringin, durum wheat bran, and mushroom myceliated grains. The increased TAC is commonly evaluated through assays such as ferric reducing antioxidant power (FRAP) or radical scavenging activity methods like DPPH and ABTS (Serra et al., 2021). The transfer of dietary polyphenols and their antioxidant metabolites into milk is considered a principal factor contributing to this augmented antioxidant status, which not only benefits the consumer but may also enhance the oxidative stability of milk and its derived products, potentially extending shelf life and preserving quality (Serra et al., 2021).

### 8.2 Milk fatty acid profile

Dietary polyphenols can significantly influence the fatty acid profile of milk fat, particularly affecting saturated fatty acid (SFA) and medium-chain fatty acid (MCFA) contents, as supported by various studies and meta-analyses (Harun et al., 2025). A primary mechanism by which certain polyphenols modify milk fat composition is through the partial inhibition of ruminal biohydrogenation, a microbial process that saturates dietary unsaturated fatty acids (Serra et al., 2021). By limiting this transformation, some polyphenols enhance the transfer of beneficial polyunsaturated fatty acids (PUFAs) into milk. For example, feeding red clover silage, rich in isoflavones and active polyphenol oxidase (PPO), has been associated with increased concentrations of favorable C18 PUFAs, such as α-linolenic acid, in milk (Lee, 2014). PPO activity leads to the formation of protein-bound polyphenols, which are thought to protect lipids from lipolysis and subsequent biohydrogenation. Similarly, supplementation with grape residue silage has been reported to raise PUFA levels in cow milk (Serra et al., 2021). Dietary administration of Olea europaea L. (olive) extract resulted in milk with decreased SFA and increased PUFAs, including omega-6 and omega-3 fatty acids (Di Meo et al., 2023). Tannins have also been investigated for their capacity to modulate ruminal biohydrogenation pathways. Some studies indicate that tannins may alter rumen microbial populations or enzymatic activities involved in biohydrogenation, potentially increasing the accumulation of intermediates such as vaccenic acid (VA, *trans*-11 C18:1) and conjugated linoleic acid (CLA, particularly rumenic acid, *cis*-9, *trans*-11 C18:2) in milk (Birkinshaw et al., 2020). Nevertheless, effects of tannins on milk fatty acid profiles are variable and depend on tannin type, concentration, and the basal diet. These alterations—namely, reduced SFAs and increased PUFAs such as omega-3 fatty acids and CLA—are generally viewed as nutritionally advantageous for human health, highlighting an important indirect mechanism by which dietary polyphenols enhance the health-promoting properties of milk.

### 8.3 Milk protein and lactose content

The influence of polyphenols supplementation on major milk solids—protein and lactose—is less consistent and appears highly dependent on the polyphenols source, type, dosage, and dietary context (Harun et al., 2025). A meta-analysis revealed that polyphenols from agro-industrial by-products significantly impacted milk protein content, whereas those derived from grains significantly affected milk lactose content (Harun et al., 2025). For instance, coix seed extract was reported to induce a quadratic increase in milk protein content in goats with rising supplementation levels (Harun et al., 2025). Supplementation with Olea europaea L. extract has also been shown to increase both milk protein and lactose concentrations in dairy cows (Aguiar et al., 2014). Furthermore, a meta-analysis on flavonoid supplementation in cattle diets concluded that flavonoids generally elevate milk protein content (Orzuna-Orzuna et al., 2023). This variability suggests that different polyphenols or associated bioactive compounds in supplemented feeds may exert distinct physiological effects, either by modifying ruminal microbial protein synthesis or volatile fatty acid (VFA) production—thereby affecting energy availability for lactose synthesis—or through systemic effects on mammary gland synthetic pathways.

### 8.4 Somatic cell count (SCC)

Milk somatic cell count (SCC) is a key indicator of udder health, with lower SCC values generally signifying healthier mammary tissue and higher-quality milk. Some polyphenols supplements have demonstrated potential to reduce SCC. For example, a meta-analysis of dietary flavonoid supplementation in cattle reported a significant decrease in milk SCC (Orzuna-Orzuna et al., 2023). This effect is likely attributable to the anti-inflammatory and antioxidant properties of flavonoids, which may mitigate inflammatory responses in the mammary gland and consequently improve udder health.

### 8.5 Sensory properties of milk and dairy products

The transfer of polyphenols into milk can sometimes alter sensory attributes, including flavour, color, taste, and aroma, of the milk or their processed dairy products, although such effects are not consistently observed and depend on the type and concentration of polyphenols present (Serra et al., 2021). For example, Caciotta cheese produced from milk of cows fed diets supplemented with dried grape pomace was reported to exhibit a darker color, firmer texture, and less sweet flavor compared to control cheese (Serra et al., 2021). Conversely, another study involving stirred yogurt fortified with pomegranate peel extracts found no significant differences in sensory attributes such as flavor, body, texture, or appearance (Serra et al., 2021). These findings indicate that sensory modifications are possible but appear specific to the polyphenols source and transferred compound concentrations and warrants further investigation.

In summary, dietary polyphenols can elicit a variety of effects on milk quality that extend beyond their direct metabolite presence. They enhance antioxidant capacity, beneficially modulate the fatty acid profile—primarily through protection of PUFAs in the rumen—and variably influence protein, lactose, and SCC. These multifaceted impacts underscore the potential of polyphenols supplementation to improve both the nutritional and technological attributes of milk.

### 8.6 Implications for precision feeding and nutraceutical strategies

The evidence reviewed in this article suggests that dietary polyphenols can be strategically used within precision feeding and nutraceutical frameworks in dairy systems. By exploiting specific polyphenol classes and sources—such as tannin-containing forages and extracts, grape and citrus by-products, cocoa bean shell, tea-derived polyphenols and other phytogenic additives—it is possible to target distinct functions, including modulation of rumen fermentation and methane output, nitrogen partitioning, oxidative and inflammatory status, and milk quality traits (Ahmed et al., 2024; Bešlo et al., 2022; Besharati et al., 2022; Di Meo et al., 2023; Formato et al., 2022; Jalal et al., 2025; Olagaray & Bradford, 2019; Ponnampalam et al., 2022; Renna et al., 2022; Serra et al., 2021; Tzamaloukas et al., 2021; Wanapat et al., 2024). Within a precision feeding perspective, these compounds could be used to tailor rations according to production goals (e.g., methane mitigation, enhanced antioxidant status, modified milk fatty acid profile) and animal characteristics, while taking into account rumen microbial “metabotypes” and lactation stage. However, the marked variability in feed-to-milk transfer efficiencies, incomplete knowledge of dose–response relationships and limited standardisation of analytical and experimental approaches still hamper the design of fully predictive nutraceutical interventions.

## 9 Potential human health implications of consuming polyphenols-enriched milk

The enrichment of bovine milk with polyphenols or their metabolites through dietary strategies in dairy cows has attracted considerable interest, largely due to the prospective health benefits these bioactive compounds may offer to human consumers. While milk is inherently a source of essential nutrients, its enhancement with polyphenols could further augment its status as a functional food with added health-promoting properties (Shivashankara & Acharya, 2010). When considering potential human health implications of consuming polyphenols-enriched milk, it is therefore essential to keep in mind the “bioavailability–bioactivity paradox” discussed in Section 6.x. The biological effects of such products are likely to depend not only on the absolute concentrations of a few marker metabolites in milk, but also on the broader pattern of microbial- and host-derived metabolites, their conjugation status and interactions with other milk components.

### 9.1 Enhanced antioxidant intake

Milk naturally enriched with polyphenols compounds, and thereby exhibiting increased total antioxidant capacity (TAC), may contribute significantly to the overall dietary antioxidant intake of consumers (Serra et al., 2021). Antioxidants are crucial in neutralizing reactive oxygen species (ROS) and other free radicals within the body, protecting cellular structures and tissues from oxidative damage (Ponnampalam et al., 2022). Chronic oxidative stress is implicated in the etiology of various non-communicable diseases, including cardiovascular diseases, neurodegenerative disorders, and certain cancers. Consequently, the consumption of foods such as polyphenols-enriched milk, which potentially elevate antioxidant intake, is hypothesized to confer protective health effects.

### 9.2 Specific bioactivities of milk-transferred metabolites

Beyond generalized antioxidant effects, particular polyphenols metabolites transferred into milk have been linked to distinct biological activities demonstrated in vitro and in vivo (animal) models:

• **Equol:** This microbial metabolite of isoflavones (daidzein and genistein), present in milk from cows consuming soy or red clover, exhibits greater estrogenic and antioxidant activities than its parent compounds (Křížová et al., 2021). Due to its structural similarity to estradiol, equol can bind estrogen receptors and may modulate hormone-dependent physiological processes. It has been explored for potential roles in preventing cardiovascular diseases, osteoporosis, and hormone-related cancers such as breast and prostate cancer (Gong et al., 2023). Notably, consumption of soy milk, a direct source of isoflavones metabolized to equol by gut microbiota, has been associated with reductions in LDL cholesterol (Yuan et al., 2007). The presence of pre-formed equol in bovine milk could provide these benefits more directly, particularly for individuals with gut microbiota that inefficiently produce equol.
• **Urolithins:** Derived from ellagitannins—found in foods such as pomegranates, berries, walnuts, and certain forages—and their precursor ellagic acid, urolithins (A, B, and C) have been detected in commercial cow’s milk and are produced endogenously in cattle (González-Barrio et al., 2012). In vitro and in vivo (animal) studies have highlighted their broad biological activities, including anti-inflammatory, anticarcinogenic, antioxidant, and antimicrobial effects (Espín et al., 2013). If transferred in sufficient quantities to milk, these metabolites may contribute to such health-protective actions in consumers.
• **Phenyl-γ-valerolactones (PVLs):** Major microbial metabolites of flavan-3-ols, abundant in tea, cocoa, grapes, and apples, PVLs have been identified in commercial bovine milk (Mena et al., 2019). Emerging evidence suggests PVLs may exert anti-inflammatory, neuroprotective, and cardioprotective effects (Mena et al., 2019), presenting an additional route by which dairy products could deliver bioactive polyphenols derivatives.
• **Phenolic Acids:** Simpler phenolic acids and their derivatives—either transferred directly into milk or formed as breakdown products of complex polyphenols—also contribute to bioactivity. For instance, 3,4-dihydroxyphenylacetic acid (3,4-DHPAA), a metabolite of quercetin, has demonstrated protective effects on pancreatic β-cells in experimental models (Carrasco-Pozo et al., 2015). Metabolites of common dietary phenolic acids such as caffeic and ferulic acids similarly retain notable antioxidant properties (Carrasco-Pozo et al., 2015; Lafay & Gil-Izquierdo, 2008).

The potential health benefits of these metabolites extend beyond their antioxidant capacity, involving interactions with specific cellular signaling pathways. For example, equol’s modulation of estrogen receptors (Gong et al., 2023) and urolithins’ influence on inflammatory pathways (Espín et al., 2013) represent targeted biological effects that may be physiologically relevant even at the relatively low concentrations typically found in milk.

### 9.3 Studies on polyphenols-enriched milk

Although the in vitro bioactivities of individual polyphenols metabolites are promising, direct evidence from human studies evaluating the health effects of consuming milk enriched through dairy cow dietary manipulation remains limited.

• **Animal Models:** Several animal studies have reported positive outcomes. For example, diabetic rats fed milk naturally enriched with both polyunsaturated fatty acids (PUFAs) and polyphenols—derived from cows supplemented with flaxseed oil, a propolis-based product, and vitamin E—demonstrated attenuated metabolic disturbances, including reduced LDL cholesterol, improved glucose tolerance, and enhanced body composition (increased muscle mass and decreased mesenteric fat) compared to rats fed control milk or milk enriched solely with PUFA (Santos et al., 2017).
• **Human Studies:** Clinical trials directly assessing health outcomes following consumption of milk from cows fed specific polyphenols-enriched diets are scarce. Much of the current understanding is extrapolated from studies on direct human intake of polyphenols-rich foods (e.g., fruits, vegetables, soy), isolated supplements, or in vitro investigations (Fiecke et al., 2025). For example, although casein micelles in milk have been shown to transport polyphenols and this has been associated with potential antiproliferative effects against colon cancer cells in vitro (Khan et al., 2019), such findings do not conclusively demonstrate benefits from milk produced by polyphenols-supplemented cows. Furthermore, human research on polyphenols in milk has predominantly focused on maternal milk and infant development following maternal intake of polyphenols-rich diets, rather than on bovine milk enhanced through animal feeding strategies (Fiecke et al., 2025). These indicate dietary polyphenols effects on milk polyphenols concentration and their impact on milk preservative- and nutritional-aspects as well as human health upon consumption are inconclusive and need further investigations.

This gap in direct clinical evidence constitutes a significant barrier to substantiating health claims for polyphenols-enriched bovine milk. Rigorous, targeted human intervention studies are essential to validate the hypothesized benefits.

### 9.4 Considerations for bioavailability from milk

The ultimate health impact of polyphenols metabolites in enriched milk is critically dependent on their bioavailability in humans. The chemical forms of these compounds in milk—often as glucuronide or sulfate conjugates—and interactions with the milk matrix itself can influence their absorption and subsequent physiological effects (Shivashankara & Acharya, 2010). Interestingly, some evidence suggests the milk matrix may enhance the intestinal absorption of certain polyphenols; for example, in vitro studies indicate that milk proteins can improve the absorption of green tea catechins (Xie et al., 2013). Nevertheless, it is important to recognize that the concentrations of many specific polyphenols metabolites in milk are generally low, often in the nanogram per milliliter range as discussed previously. While these levels may contribute to the overall antioxidant properties of milk, the systemic dose of any single metabolite obtained via milk consumption is likely modest compared to direct intake of polyphenols-rich plant foods (Shivashankara & Acharya, 2010). Therefore, observable health benefits may require sustained, long-term consumption of such enriched milk or depend on the presence of highly bioactive metabolites and synergistic interactions among multiple compounds within the milk matrix.

## 10 Gaps in knowledge and future research directions

Despite substantial progress in elucidating the effects of dietary polyphenols on dairy cattle physiology and milk composition, significant knowledge gaps remain. Addressing these gaps is essential for optimizing supplementation protocols, ensuring consistent milk enrichment, and conclusively establishing benefits for both animal health and human consumers. A central challenge within this field lies in managing and understanding the inherent variability associated with polyphenols sources, the complexity of rumen microbial ecosystems, individual animal metabolic responses, and analytical measurement techniques. Future research should systematically dissect these sources of variation to develop more predictable and reliable strategies for enhancing milk quality via dietary polyphenols.

Beyond the need for additional intervention trials, there is a clear lack of mechanistic studies that integrate rumen microbial transformations, host conjugation pathways and mammary transport processes into a coherent framework. Most current evidence addresses these steps in isolation—either focusing on rumen fermentation, systemic pharmacokinetics or mammary efflux transporters such as ABCG2 (García-Lino et al., 2019; Gong et al., 2024; Mahnke et al., 2016; Otero et al., 2015)—which hampers quantitative predictions of how specific dietary interventions will translate into milk polyphenols profiles and downstream functional effects.

### 10.1 Dose-response relationships

Although several studies have investigated the effects of varying polyphenols dosages, comprehensive dose-response relationships between the intake of specific polyphenols sources or purified compounds by dairy cows and the resulting concentrations of targeted bioactive metabolites in milk remain incomplete or insufficiently characterized (Serra et al., 2021). Many studies have employed single dosage levels or a narrow range of supplementation, limiting the ability to determine optimal doses that maximize desirable metabolite transfer without negatively affecting animal performance or feed intake. The available literature and the impact of dietary polyphenols on milk polyphenols or their conjugated derivatives concentration and subsequent effects on milk preservation, nutritional value and human health are inconclusive. Future investigations should incorporate broader dosage ranges and examine potential non-linear or threshold effects.

### 10.2 Long-term effects

Most existing intervention studies are relatively short-term in duration. There is a pressing need for research into the long-term consequences of sustained polyphenols supplementation on dairy cow health, productive lifespan, reproductive performance, and the stability of milk polyphenols enrichment across multiple lactation cycles. Additionally, the potential adaptation of the rumen microbiota to chronic polyphenols exposure and its implications for metabolite production and transfer warrant thorough investigation.

### 10.3 Influence of animal factors

Intrinsic animal factors that influence polyphenols metabolism and transfer into milk require further detailed study:

• **Lactation Stage and Parity:** Given their well-documented effects on overall milk composition and cow physiology (Connolly et al., 2023), it is highly plausible that lactation stage and parity significantly modulate the transfer efficiency of key polyphenols metabolites such as equol, urolithins, and valerolactones. However, empirical data quantifying these influences remain scarce (Rashidinejad, 2015). A better understanding of these variables is crucial to achieving consistent milk quality.
• **Host Genetics and Rumen Microbiome:** The role of host genetics, including potential “metabotypes,” and the extensive inter-individual variability in rumen microbiome composition and function in determining the profile and concentration of polyphenols metabolites in milk, requires further elucidation (González-Barrio et al., 2012). Identification of specific microbial taxa or functional pathways responsible for the production of desirable metabolites may enable targeted microbiome modulation strategies to enhance milk bioactivity.

### 10.4 Mechanisms of mammary gland transport

The precise molecular mechanisms governing the transport of various polyphenols metabolites—including aglycones and their conjugated derivatives—across the blood-mammary barrier into milk remain largely unknown (Bešlo et al., 2022). Characterization of the specific transporters involved, such as members of the ATP-binding cassette (ABC) or solute carrier (SLC) families, as well as the regulatory factors influencing their activity, would provide valuable insights for potentially enhancing the secretion of bioactive compounds into milk.

### 10.5 Bioavailability and bioactivity of milk-derived polyphenols in humans

A critical gap exists regarding the actual bioavailability and subsequent physiological effects of polyphenols metabolites derived from enriched bovine milk upon human consumption. While in vitro and animal model data have begun to accumulate, robust human clinical trials are indispensable to substantiate health claims associated with these specialized polyphenols-enriched dairy products (Khan et al., 2019).

### 10.6 Interaction effects

• **Polyphenols-Diet Interactions:** The complex interactions between different dietary polyphenols themselves, as well as between polyphenols and other major dietary components such as fiber, fat, and protein types, influence rumen metabolism, systemic bioavailability, and transfer into milk. These interactions remain incompletely understood and merit further investigation (Bohn, 2014).
• **Forage Polyphenols Oxidase (PPO) Influence:** The role of forage PPO activity, particularly regarding the formation of protein-bound phenols, in modulating the bioavailability and transfer of non-substrate polyphenols (i.e., those not directly oxidized by PPO) or their metabolites into milk is poorly characterized (Lee, 2014). It is plausible that PPO-induced alterations of the forage matrix indirectly affect other phenolic compounds and their subsequent bioactivity.

### 10.7 Standardization of methodologies

To enhance comparability and reliability across studies, there is an urgent need for greater standardization of analytical methodologies employed in the quantification of polyphenols metabolites within complex matrices such as feed and milk (Daems et al., 2016). Furthermore, the development and wider commercial availability of analytical reference standards for a broader spectrum of polyphenols metabolites, particularly conjugated forms and specific microbial degradation products, are critical to advancing research in this field (González-Barrio et al., 2012). Adopting holistic, systems-biology research approaches would be highly advantageous. Integrating comprehensive data sets encompassing feed composition, rumen metagenomics and metabolomics, host metabolomics (including plasma and milk), and ultimately human physiological responses to consumption of enriched dairy products, would provide a more complete understanding of the intricate pathways from feed to health outcomes. Such approaches can help identify critical control points and optimize strategies for achieving desired milk enrichment.

Finally, alongside scientific progress, the economic feasibility and practical implementation of large-scale polyphenols supplementation within the dairy industry must be carefully evaluated. Techno-economic assessments should accompany research efforts to ensure that promising interventions are viable and adoptable by dairy producers (Serra et al., 2021).

## 11 Conclusions

The incorporation of polyphenol-rich feedstuffs and extracts into the diets of dairy cattle has been shown to significantly influence milk composition, particularly with respect to its polyphenols compounds profile. Dietary polyphenols undergo extensive metabolic transformation within the ruminant system, with the rumen microbiota playing a central role in hydrolyzing glycosidic bonds, degrading complex polyphenols structures, and generating a diverse array of smaller, often more bioavailable metabolites. Among the key classes of metabolites identified as transferable into bovine milk are isoflavones and their derivatives, such as equol (notably derived from soy and red clover), flavan-3-ol metabolites including phenyl-γ-valerolactones (originating from sources such as grape seed extract), ellagitannin metabolites like urolithins (from tannin-rich plants including oak and pomegranate by-products), as well as various simpler phenolic acids, with hippuric acid frequently detected at relatively high concentrations. The extent of metabolite transfer and the specific polyphenols profile present in milk are highly variable, reflecting a complex interplay of factors. These include the chemical nature, structural characteristics, and dosage of ingested polyphenols, interactions within the feed matrix, and multiple animal-specific variables such as the composition and activity of the rumen microbiota, host genetic factors (metabotypes), physiological status (including lactation stage and parity), and overall health condition. Although the precise mechanisms underlying the transport of these metabolites across the blood-mammary barrier remain incompletely understood, evidence suggests involvement of both passive diffusion and active carrier-mediated transport processes. In addition to modulating the polyphenols metabolite content, dietary supplementation with polyphenols-rich sources can confer other notable improvements to milk quality, particularly through enhanced total antioxidant capacity and beneficial modifications to the fatty acid profile—often achieved by protecting polyunsaturated fatty acids from ruminal biohydrogenation. Effects on macronutrient composition, including protein and lactose content, appear to be more variable and dependent on the specific polyphenols source and supplementation regimen. While the concept of producing “functional milk” enriched with bioactive polyphenols metabolites holds considerable promise, supported by in vitro and in vivo (animal) models evidence for certain compounds, direct evidence of tangible human health benefits from consuming polyphenols-enriched bovine milk at current metabolite concentrations remains limited. Many metabolites are present at relatively low levels, indicating that meaningful health impacts may require either highly potent individual metabolites, synergistic interactions among multiple compounds, or sustained long-term consumption. Future research should prioritize the elucidation of dose-response relationships, the influence of animal-specific factors on metabolite production and transfer, and a detailed understanding of mammary gland transport mechanisms. The standardization of analytical methods and the development of comprehensive reference standards for polyphenols metabolites are essential for advancing this field. Moreover, the strategic utilization of agro-industrial by-products as sustainable and economically viable sources of dietary polyphenols offers considerable potential benefits for the dairy industry.

Beyond summarising the current evidence along the “from rumen to milk” continuum, the available data clearly show that our understanding of polyphenol-derived metabolites in dairy cows and milk is still fragmentary. Several research priorities emerge from this critical analysis.

Future studies should provide a more complete and quantitative characterisation of polyphenol-derived metabolites in milk, distinguishing between free, conjugated and protein-bound forms, and covering microbial as well as host-generated species (e.g., urolithins, phenyl-γ-valerolactones, phenolic acids, equol, enterolactone). Advanced LC–MS-based workflows developed for milk metabolomics and human milk studies offer promising tools in this regard. Stable isotope-labelled polyphenols (or polyphenol-rich feeds) should be used more systematically to quantify metabolic fluxes and true feed-to-milk transfer efficiencies, linking rumen transformations, post-ruminal absorption, systemic circulation and mammary secretion within a single experimental framework. Such studies would greatly refine current estimates of bioavailability and help to resolve the bioavailability–bioactivity paradox described in this review. Integrative designs combining rumen microbiome characterisation, host transcriptomics and proteomics with targeted or untargeted milk metabolomics are needed to connect dietary interventions, microbial metabotypes, mammary transport processes (e.g., ABCG2/BCRP activity) and changes in the milk metabolome. Systems biology and modelling approaches could further support prediction of feed-to-milk responses under practical conditions. Finally, there is a need for more standardised intervention designs (including well-defined dietary formulations, doses and time frames), harmonised analytical panels and explicit evaluation of functional endpoints related to animal health, milk quality and technological properties, as well as, where feasible, consumer-relevant outcomes. Linking molecular-level changes to robust functional readouts will be essential to fully exploit dietary polyphenols in precision feeding strategies and in the development of genuinely functional dairy products. Taken together, addressing these research needs will be crucial to move from descriptive evidence towards predictive and mechanistically informed use of dietary polyphenols in dairy cows and their milk. From an applied perspective, addressing these research gaps will be essential to move from empirical use of polyphenol-rich feeds towards truly precision feeding and nutraceutical strategies in dairy cows, where specific polyphenol classes, doses and feed matrices are selected to achieve defined metabolic, environmental and product-quality outcomes with predictable feed-to-milk responses.

## Ethical statement

Not applicable.

## CRediT authorship contribution statement

**Lucrezia Forte:** Writing – original draft, Methodology, Investigation. **Nives Parabita:** Writing – original draft, Investigation. **Marta Santoro:** Writing – original draft, Investigation. **Francesco Longobardi:** Writing – review & editing. **Giuseppe Natrella:** Writing – review & editing, Visualization. **John Quiñones:** Writing – review & editing, Visualization. **Eric N. Ponnampalam:** Writing – review & editing, Visualization. **Igor Tomasevic:** Writing – review & editing. **Pasquale De Palo:** Writing – review & editing. **Aristide Maggiolino:** Writing – original draft, Supervision, Methodology, Conceptualization.

## Declaration of competing interest

Given his role as Editor in Chief, Eric Ponnampalam had no involvement in the peer-review of this article and has no access to information regarding its peer-review. Full responsibility for the editorial process for this article was delegated to another journal editor.
